# Mutations Suppressing the Lack of Prepilin Peptidase Provide Insights Into the Maturation of the Major Pilin Protein in Cyanobacteria

**DOI:** 10.3389/fmicb.2021.756912

**Published:** 2021-10-12

**Authors:** Markéta Linhartová, Petra Skotnicová, Kaisa Hakkila, Martin Tichý, Josef Komenda, Jana Knoppová, Joan F. Gilabert, Victor Guallar, Taina Tyystjärvi, Roman Sobotka

**Affiliations:** ^1^Institute of Microbiology of the Czech Academy of Sciences, Prague, Czechia; ^2^Faculty of Science, University of South Bohemia, České Budějovice, Czechia; ^3^Biotechnology/Molecular Plant Biology, University of Turku, Turku, Finland; ^4^Barcelona Supercomputing Center, Barcelona, Spain; ^5^ICREA: Institucio Catalana de Recerca i Estudis Avançats Passeig Lluis Companys, Barcelona, Spain

**Keywords:** Type IV pili, *Synechocystis*, photosystem II, PilD peptidase, suppressor mutations

## Abstract

Type IV pili are bacterial surface-exposed filaments that are built up by small monomers called pilin proteins. Pilins are synthesized as longer precursors (prepilins), the N-terminal signal peptide of which must be removed by the processing protease PilD. A mutant of the cyanobacterium *Synechocystis* sp. PCC 6803 lacking the PilD protease is not capable of photoautotrophic growth because of the impaired function of Sec translocons. Here, we isolated phototrophic suppressor strains of the original Δ*pilD* mutant and, by sequencing their genomes, identified secondary mutations in the SigF sigma factor, the γ subunit of RNA polymerase, the signal peptide of major pilin PilA1, and in the *pilA1-pilA2* intergenic region. Characterization of suppressor strains suggests that, rather than the total prepilin level in the cell, the presence of non-glycosylated PilA1 prepilin is specifically harmful. We propose that the restricted lateral mobility of the non-glycosylated PilA1 prepilin causes its accumulation in the translocon-rich membrane domains, which attenuates the synthesis of membrane proteins.

## Introduction

Type IV pili are appendages on the cell surface that belong to the most versatile prokaryotic nano-machines mediating diverse functions including adhesion, aggregation, motility, secretion or DNA uptake (Giltner et al., 2012). The key building block of the Type IV pili is a so-called major pilin protein, which is synthesized as an integral cell membrane protein but later is extracted from the membrane bilayer and assembled into long helical polymers protruding from the cell. In Gram-negative bacteria, the assembly (and disassembly) of pili is driven by ATP hydrolysis and requires large molecular machinery anchored in the cytoplasmic and outer membranes, spanning the whole periplasmic space (Craig et al., 2019).

The highly conserved feature of Type IV pilins is their process of maturation. Pilin monomers are produced as precursors called prepilins with an N-terminal signal sequence required for the insertion of prepilins into the membrane followed by a conserved transmembrane segment (Giltner et al., 2012). The N-terminal signal segment is then removed by the integral membrane peptidase PilD that cleaves prepilin roughly proximal to the cytoplasmic membrane surface (Strom et al., 1993; LaPointe and Taylor, 2000). Analysis of various bacterial species lacking the PilD peptidase has demonstrated that removal of the signal peptide is essential for pilus assembly (Goosens et al., 2017); non-matured prepilins remain anchored in the membrane (Strom et al., 1993). Also, *O*-glycosylation of major pilins, reported in many bacterial strains, probably facilitates pilus assembly or its stability (Marceau et al., 1998).

Cyanobacteria are a unique group of Gram-negative bacteria with the ability to perform oxygenic photosynthesis and fix carbon dioxide into organic compounds. In contrast to other prokaryotes, cyanobacteria contain an endogenous membrane system called thylakoid membranes (TM), which is very abundant in photosystem I and photosystem II (PSII) complexes—large, membrane-embedded, oxidoreductases powered by light. Photosystems are essential for all oxygenic phototrophs as they use the energy of photons to generate highly oxidizing/reducing species that are needed for the oxidation of water and the reduction of NADP^+^. Although cyanobacteria evolved this metabolic strategy not used by other prokaryotes, they possess many typical bacterial structures including Type IV pili (Schuergers and Wilde, 2015).

In our previous study, we revealed an intimate and unexpected link between the maturation of pilins and the biogenesis of photosynthetic membrane complexes (Linhartová et al., 2014). We found that the elimination of the PilD protease and the consequent accumulation of prepilins in the cyanobacterium *Synechocystis* sp. PCC 6803 (hereafter *Synechocystis*) triggered degradation of the SecY translocase and the YidC insertase. The latter protein is known to associate with the SecYEG heterotrimer forming a component of the bacterial holo-translocon—molecular machinery promoting the insertion of most membrane proteins into the lipid bilayer (Sachelaru et al., 2017). Disturbing the holo-translocon drastically reduces the synthesis of membrane proteins including all core subunits of PSII (Linhartová et al., 2014). Due to the toxicity of prepilins, the Δ*pilD* strain was no longer able to grow photoautotrophically though proliferation under mixotrophic conditions (in the presence of glucose) was possible (Linhartová et al., 2014). Interestingly, deletion of the *pilA1* gene coding for the major PilA1 pilin completely rescued the photosynthetic capacity of the Δ*pilD* strain (Linhartová et al., 2014). It led us to a prediction that spontaneous suppressor mutations reducing the synthesis/accumulation of PilA1 prepilin (pPilA1), or its harmful impact on the activity of translocons, may restore the photoautotrophic growth of the Δ*pilD* mutant.

Here, we identified four different photoautotrophic Δ*pilD* suppressor (revertant) strains. One revertant strain contains a mutation in the SigF sigma factor and the other one is a double mutant containing point mutations in the SigF factor and the γ subunit of the RNA polymerase (RNAP). Two other suppressor lines have mutations in the *pilA1* gene or the *pilA1-pilA2* intergenic region. Effects of these mutations as well as the suppressor effect of mixotrophic growth conditions were analyzed in detail. Our results suggest that there is a link between the PilA1 prepilin (pPilA1) toxicity and the lagged glycosylation of this protein. We propose that the accumulation of the non-glycosylated pPilA1 in the biosynthetically active membrane zones is responsible for the growth arrest of the Δ*pilD* mutant.

## Materials and Methods

### *Synechocystis* Strains and Cultivation

Strains used in this study were derived from the *Synechocystis* GT-W substrain, Δ*pilD* and Δ*pilA1*/Δ*pilA2* (Δ*pilA1*/*2*) mutant strains were described in Linhartová et al. (2014) and the *sigF*^–^ mutant in Barker et al. (2006). Inactivation of the *pilA2* gene by insertion of a Spectinomycin resistance gene has been performed by Bhaya et al. (2000). For this work, the identical mutation was fully segregated in the GT-W substrain (*pilA2*^–^ strain; for this work “–”superscript is used for insertion mutants). The Δ*pilD*/*pilA2*^–^ mutant was described in Linhartová et al. (2014). Photoautotrophic suppressor mutants of the Δ*pilD* strain were generated by cultivation on a BG-11 plate with 5 mM glucose for 2 weeks at normal light intensity (40 μmol of photons m*^–^*^2^ s*^–^*^1^) at 28°C. For mixotrophic conditions (Glc^+^), the BG-11 medium was supplemented with 5 mM glucose. Liquid cultures were grown in Erlenmeyer flasks under normal light at 28°C on a rotary shaker (120 rpm). Mixotrophic cultures were diluted daily with fresh Glc^+^ medium and collected at OD_730 nm_ of ∼ 0.4. To study the effects of photoautotrophic conditions (Glc*^–^*), the cells grown in Glc^+^ conditions were harvested at 6000 rpm, washed with BG-11 medium, diluted into BG-11 medium without glucose and then incubated at normal light for 48 h. To determine optical density of aggregated strains, cell aggregates were disintegrated using an IKA TP 18/10 Ultra-Turrax homogenizer (IKA Werke, Germany).

### Whole-Genome Re-Sequencing and Variant Detections

DNA for genomic sequencing was isolated as described by Ermakova-Gerdes and Vermaas (1999), with slight modifications. Briefly, the cells were washed by saturated NaI solution and lysed by lysozyme and SDS. The lysate was treated with proteinase K, extracted with phenol, phenol-chloroform (1:1) and chloroform and treated with RNase. The DNA was precipitated with sodium acetate and ethanol, air-dried and resuspended in water. *Synechocystis* re-sequencing was performed commercially at the Gene Profiling Facility, Princess Margaret Hospital, Toronto. 100 bp paired-end sequencing was performed on an Illumina HiSeq 2000 platform. The average sequencing depth was around 70. Reads were mapped to the *Synechocystis* GT-S sequence (NC_017277) and the variants were called using default settings of the Geneious 11.0 software.^1^ Only variants with a higher than 60% frequency were considered.

### Extraction of RNAs and Northern Blotting

Total RNA was isolated from cells (40 mL cultures at OD_750 nm_ ∼ 0.5) using a hot phenol method according to Tyystjärvi et al. (2001), except that cell pellets were resuspended in 0.3 M sucrose and 10 mM sodium acetate (pH 4.5). Samples containing 3.75 μg total RNA were denatured with glyoxal and separated on a 1.2% agarose gel in phosphate buffer and transferred to Hybond-N membrane (Tyystjärvi et al., 2001). The *pilA1* and 16S rRNA genes were amplified from genomic DNA using *pilA1-f/pilA1-r* and *rrn-f*/*rrn-r* primers, respectively (Supplementary Table 1). DNA fragments were labeled with α-dCTP 10 mCi/mL (Perkin Elmer) using the Prime-a-Gene Labeling System (Promega, United States) according to the manufacturer’s instructions. The probes were purified using Illustra ProbeQuant G-50 Micro Columns (GE Healthcare). Membranes were prehybridized in 6 × SSC, 1 × Denhardt’s, 0.1% SDS, 100 μL/mL herring sperm DNA at 67°C for 1 h. A denatured *pilA* probe was added and hybridized at 67°C overnight. Membranes were rinsed twice with 3 × SSC, 0.1% SDS and then washed twice for 10 min at 60°C in 3 × SSC, 0.1% SDS and autoradiographed. The *pilA* probe was removed by washing the membrane three times with 0.5% SDS at 95°C for 10 min and then reprobed with the 16S rRNA probe.

### Protein Isolation, Electrophoresis, Immunoblotting, and Co-Immunoprecipitation

About 100 mL of cells at an OD_730 nm_ of ∼0.4 were harvested by centrifugation at 6,000 × *g* for 10 min at 4°C and pelleted cells were first washed with and then resuspended in buffer A (25 mM MES/NaOH, pH 6.5, 10 mM MgCl_2_, 10 mM CaCl_2_, and 25% glycerol). Cells were mixed with 100–200 μm diameter glass beads in 1:1 ratio (1 volume of dense cell solution with 1 volume of glass beads) and broken (6 × 40 s) using a MiniBeadbeater 16 (Biospec, United States). To separate soluble and membrane fractions, samples were centrifuged at 30,000 × *g* for 15 min at 4°C. Pelleted membranes were washed once with an excess of buffer A and then resuspended in 250 μL of buffer A. Chlorophyll (Chl) concentration in the isolated membranes was measured spectrophotometrically according to Porra et al. (1989).

To detect the amount of PilA1 protein by immunoblotting, membrane proteins containing 1 μg of Chl were solubilized with 2% SDS and 1% dithiothreitol for 30 min at room temperature and separated on a 12–20% or 16–20% linear gradient SDS-PAGE gel containing 7 M urea (Komenda et al., 2019). The proteins were transferred to a PVDF membrane and the PilA1 protein was detected with a specific primary antibody raised against amino acids 147-160 of the PilA1 (prepared commercially by GenScript, United States). The antibody raised against the recombinant fragment R117-S384 of *Synechocystis* YidC was kindly provided by Prof. Jörg Nickelsen (Ludwig-Maximilians-University, Munich, Germany). The antibody against *Synechocystis* Ycf48 was kindly provided by Professor Peter Nixon (Imperial College, United Kingdom). The secondary antibody was conjugated with horseradish peroxidase (Sigma, Germany) and the signal was detected using Immobilon Crescendo substrate (Millipore, United States). Membrane proteins (each sample contained 1 or 4 μg of Chl if not indicated otherwise) were solubilized with 2% SDS and 1% dithiothreitol for 30 min at room temperature and separated on a 16–20% linear gradient SDS-PAGE gel containing 7 M urea.

Analysis of membrane proteins (contained 4 μg of Chl) under native conditions was performed by clear-native (CN) PAGE as described by Komenda et al. (2019). Individual components of protein complexes were resolved by incubating the gel stripe from the first dimension in 2% (w/v) SDS and 1% (w/v) dithiothreitol for 30 min at room temperature, and proteins were separated along the second dimension by a gradient SDS-PAGE described above. Proteins were in-gel stained by SYPRO Orange (Sigma, Germany).

Co-immunoprecipitation was performed essentially as described in Linhartová et al. (2014). Briefly, the anti-SecY antibody was incubated overnight with membrane proteins isolated from the WT, Δ*pilD* mutant and rev2 and rev3 strains grown with glucose, and then the antibody was immobilized on Protein A—Sepharose (Sigma, Germany). The resin was extensively washed and the remaining proteins were eluted in 1% SDS/dithiothreitol and 0.05% Bromophenol Blue in 25 mM Tris/sucrose buffer and separated by 12–20% linear gradient SDS-PAGE. The gel was blotted and probed with selected antibodies. The primary antibody against the *Synechocystis* SecY and SecE proteins were raised in rabbits using the synthetic peptide fragments containing amino acids 4-14 and 1-19, respectively.

### Protein Radiolabeling

For protein labeling, cell cultures containing 75 μg of Chl mL^–1^ were incubated with a mixture of [^35^S]-Met and [^35^S]-Cys (Hartmann Analytics) at moderate light intensity (40 μmol of photons m^–2^ s^–1^) at 28°C; further details of the labeling procedure are described in Dobáková et al. (2009). Two-dimensional (2D) protein separation was performed as described above. The 2D gel was stained with SYPRO Orange, blotted onto a PVDF membrane and exposed to a phosphorimager plate (GE Healthcare, Austria) overnight and scanned by Storm 860 (GE Healthcare, Austria).

### Molecular Dynamics

The model of the thylakoid membrane, fully hydrated, with ions and equilibrated for 200 ns, has been described previously (Daskalakis, 2018). The structure of pPilA1 protein was modeled using iTASSER (Yang et al., 2015) with a C-score of = −1.3. Molecular dynamics (MD) simulations using the thylakoid membrane were performed using GROMACS version 2018.2 (SoftwareX, 2015, 1–2, 19–25), the AMBER03 forcefield (Duan et al., 2003) and the SPC water model. The protein was inserted into the membrane using Schrodinger’s Maestro software, and the remaining preparation steps (solvent box generation and ion placement) were carried out with GROMACS. The system was minimized using steep descent until the maximum force in the system was below 400 kJ/mol/nm. Next, NVT equilibration was performed at 303 K for 20 ns followed by NPT equilibration at 303 K and 1 atm for 40 ns. Finally, 500 ns production simulations were performed with frames saved every 1 ns. In both equilibration phases, as well as the subsequent production simulation, the time step was 2 fs, since h-bonds were constrained using the LINCS algorithm. The temperature was controlled using a V-rescale thermostat in the NVT equilibration and Nose-Hoover thermostat in NPT and production phases. The pressure was controlled with a Parrinello-Rahman barostat. Electrostatics were taken into account using PME with 1.2 nm electrostatic and van der Waals cut-offs.

MD simulations with the POPC membrane were run using Desmond 2015.4 (Bowers et al., 2006), the OPLS2005 force field, and the SPC water model. The protein was inserted into the membrane using Schrodinger’s Maestro software, and the remaining preparation steps were performed with Desmond. The equilibration set-up was the default protocol for Desmond, consisting of (1) Brownian Dynamics simulation in NVT at 10 K with restraints on solute heavy atoms for 100 ps, (2) NVT simulation at 10 K with restraints on solute heavy atoms for 12 ps, (3) NPT simulation at 10 K with restraints on solute heavy atoms for 12 ps, (4) NPT simulation at 300 K and 1 atm with restraints on solute heavy atoms for 12 ps, (5) NPT simulations at the same conditions with no restraints for 24 ps, and (6) production NPT simulation at the same conditions with no restraints for 500 ns.

## Results

### Spontaneous Mutations Suppressing the Lack of PilD

To generate suppressor mutations restoring the photoautotrophy of the *Synechocystis* Δ*pilD* deletion mutant, we grew Δ*pilD* cells on BG11 agar plates without glucose (Glc^–^) at normal light conditions (40 μmol of photons m^–2^ s^–1^). However, cells bleached completely in a few days without forming any suppressor colonies forcing us to try a second strategy. In contrast to photoautotrophic conditions, Δ*pilD* cells grow well on agar plates supplemented with 5 mM glucose (Glc^+^ conditions) for few days but after several days the cell pigmentation turned pale (Supplementary Figure 1) and the majority of cells died in 2 weeks. Notably, many green Δ*pilD* suppressor colonies appeared after 2 weeks on Glc^+^ plates (Supplementary Figure 1) and virtually all of them were able to grow photoautotrophically when tested on Glc^–^ plates. We selected four colonies (rev1-4), that differed markedly in size and pigmentation, for an in-depth analysis.

**FIGURE 1:**
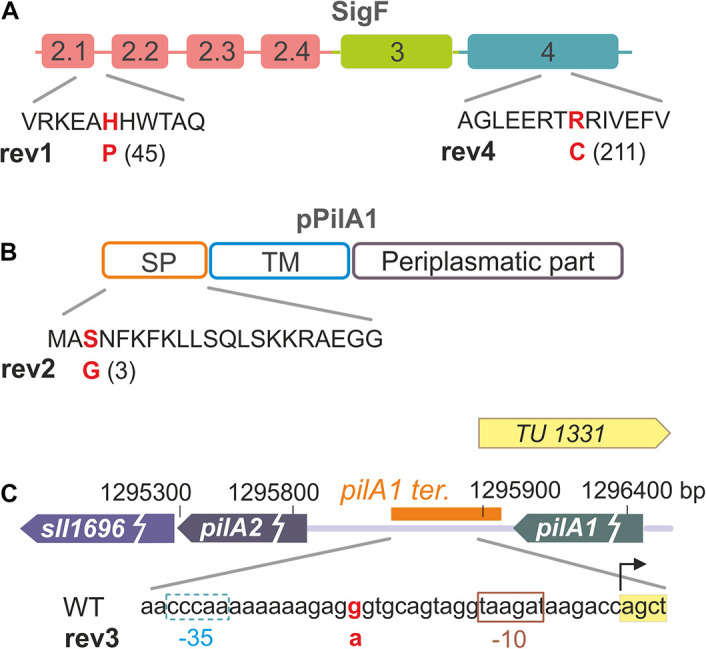
Overview of mutations that restore photoautotrophic growth of the Δ*pilD* strain. **(A)** Sigma factor SigF contains three main domains (σ2, σ3, σ4); the suppressor mutation in rev1 is located in the σ2.1 region, which interacts with the TATA box of recognized genes. The SigF mutation found in the rev4 strain is located in domain σ4, which typically interacts with the -35 segment of the promoter (see a structural model bellow). Note that the rev4 strain contains another suppressor E95K mutation in the RNAP γ subunit. **(B)** The mutation identified in the rev2 strain causes the S3G amino-acid substitution in the signal peptide of the PilA1 protein. **(C)** The rev3 strain contains a point nucleotide substitution in the intergenic region between genes coding for the pilin proteins PilA1 and PilA2. Transcription unit TU1331 including its promoter (–10 and –35 elements; Kopf et al., 2014) is depicted together with the predicted *pilA1* terminator (orange box; Hess, W. personal communication). According to Kopf et al. (2014), *pilA2* and *sll1696* genes do not have their own promoter, instead they seem to form one transcriptional unit with *pilA1*.

To identify suppressor mutations in rev1-4 strains, we performed their whole genome re-sequencing using an Illumina HiSeq platform. Obtained reads were mapped on the GT-S reference genome (Tajima et al., 2011) and, after subtraction of mutations that had been identified previously to occur in our WT laboratory substrain (GT-W; Tichý et al., 2016), we revealed mutations specific for the Δ*pilD* revertants (Figure 1 and Table 1). The point mutation in rev1 resulted in the amino-acid substitution H45P in the SigF sigma factor (Figure 1A) and the mutation in rev2 led to the S3G substitution in a signal peptide of the major pilin, PilA1 (Figure 1B). The mutation in rev3 is located in the *pilA1*-*pilA2* intergenic region (Figure 1C), specifically in the predicted terminator of the *pilA1* gene (Hess, W., personal communication). The rev4 strain contained two mutations leading to the R211C substitution in SigF (Figure 1A) and to the E95K substitution in the RNAP γ subunit.

**TABLE 1:** List of mutations identified by sequencing of Δ*pilD* revertants.

**Strain**	**Start**	**Size**	**NT change**	**AA change**	**Gene ID**	**Gene product**
rev1	3370196	1	A → C	H45P	*slr1564*	Sigma factor SigF
rev2	1296310	1	T → C	S3G	*sll1694*	Major pilin protein PilA1
rev3	1295748	1	G → A	−	*sll1694-sll1695* intergenic region	−
rev4	3370693	1	C → T	R211C	*slr1564*	Sigma factor SigF
rev4	1853201	1	G → A	E95K	*slr1265*	The γ subunit of the RNA polymerase

*The nucleotide position refers to the genomic sequence of the Synechocystis GT-S strain at NCBI BioProject: PRJDA67081.*

Particular mutations in the *pilA1* gene might directly reduce the PilA1 content, and thus lead to a formation of Δ*pilD* suppressor lines. Suppressor mutations in the *sigF* gene make also sense as the RNAP-SigF holoenzyme is responsible for the transcription of the specific regulon comprising the *pilA1* gene (Asayama and Imamura, 2008) and potentially also other *pilA* genes in *Synechocystis* (Flores et al., 2019; see section “Discussion”).

### Growth Phenotype of the Δ*pilD* Suppressor Strains

**FIGURE 2:**
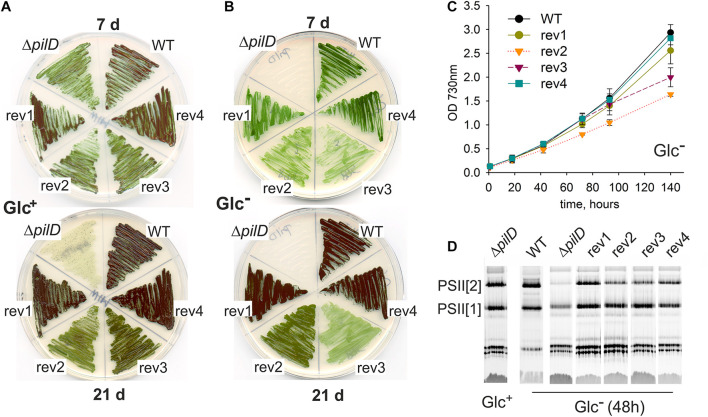
Characterization of spontaneous revertants derived from the Δ*pilD* strain. **(A)** Growth on agar plates was assessed for the WT, Δ*pilD* mutant and the four isolated Δ*pilD* revertants (rev1–4) at the 7th and 21st day of cultivation under mixotrophic conditions and **(B)** under photoautotrophic conditions. **(C)** Photoautotrophic growth of the WT and rev1-4 strains. Cells were grown in liquid culture and optical density was monitored spectroscopically. **(D)** Membrane protein complexes, isolated from the WT and mutant strains grown for 48 h in Glc^–^, were solubilized and separated by CN-PAGE. The loading corresponds to the same number of cells per lane based on OD_730_; 4 μg of Chl for WT. After separation, the gel was scanned (see Supplementary Figure 2) and PSII complexes were detected by Chl fluorescence after excitation by blue light in LAS 4000 (Fuji). PSII[1] and PSII[2] indicates the monomeric and dimeric PSII, respectively.

The rev1 and rev4 strains (suppressor mutations in the *sigF* gene) grew as well as WT on BG-11 plates both mixotrophically (Glc^+^ plates) and photoautotrophically (Glc^–^ plates; Figures 2A,B). Rev2 and rev3 strains bearing suppressor mutations in the *pilA1* gene region, grew into a much thinner layer than WT, and the rev2 strain partially bleached after 3 weeks on Glc^+^ plates (Figure 2A). Under Glc^–^ conditions, rev2 and rev3 strains proliferated only for a few first days and the rev3 mutant visibly faded showing a poorer phenotype than rev2 (Figure 2B), opposite to that seen in mixotrophic conditions. Rev1 and rev4 strains grew well also in photoautotrophic liquid culture (Glc^–^; Figure 2C). Consistent with the growth restrictions on the agar plate, both rev2 and rev3 suffered from a growth defect in Glc^–^ liquid cultures (Figure 2C). Growth of the rev2 strain was slow throughout the experiment (Figure 2C) and its Chl level remained constant (Supplementary Figure 1B), whereas the initial proliferation of rev3 was similar to WT, but at higher culture density the growth of rev3 was arrested (Figure 2C), which was accompanied by a sharp decrease in Chl content (Supplementary Figure 1B).

The fast depletion of PSII in the Δ*pilD* mutant after its shift from Glc^+^ to Glc^–^ conditions has been reported previously (Linhartová et al., 2014). We, therefore, analyzed the content of PSII in revertants 48 h after changing the growth media from Glc^+^ to Glc^–^ conditions. Indeed, Δ*pilD* mutant cells lost most of the PSII complexes and particularly the dimeric PSII form disappeared almost completely (Figure 2D). In contrast, all four suppressor mutants were capable of maintaining dimeric PSII although only rev1 and rev4 had the level comparable to WT (Figure 2D). As shown by a more detailed analysis of membrane proteins by 2D clear-native/SDS electrophoresis (CN/SDS-PAGE), rev3 cells were visibly defective in the accumulation of membrane proteins as the total levels of many of them were reduced (Supplementary Figure 2).

**FIGURE 3:**
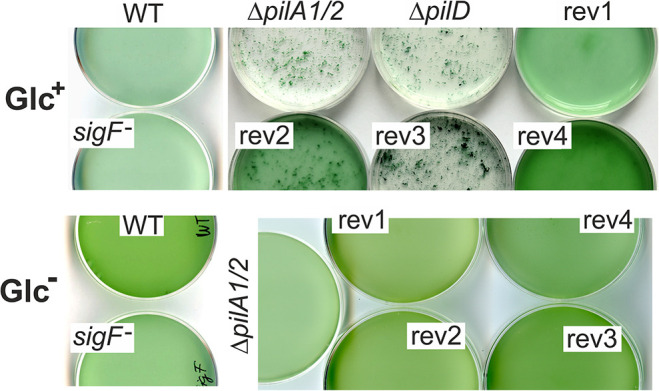
Aggregation of pilin-less cells and Δ*pilD* suppressor strains. Cell cultures were cultivated in Erlenmeyer flasks on a rotary shaker at 120 rpm in mixotrophic (Glc^+^) and photoautotrophic (Glc^–^) conditions and then poured into Petri dishes and photographed at a similar optical density (730 nm).

A striking characteristic of *Synechocystis* Δ*pilD* and Δ*pilA1/2* mutants is very intense cell aggregation under Glc^+^ growth conditions (Figure 3). This type of aggregation persists a vigorous shaking at 120 rpm and thus differs from cell flocculation characteristic of large flocs formed by cells settled down at the bottom of the cultivation flask (Conradi et al., 2019). Interestingly, the rev1 and rev4 strains, harboring mutations in the *sigF* gene, exhibited almost no tendency to form such aggregates (Figure 3) and the *sigF* deletion strain also did not aggregate (Figure 3). Aggregation of rev2 cells was weaker than that of the Δ*pilD* mutant and a portion of cells remained fully dispersed, whereas the rev3 aggregated comparably to the parental strain (Figure 3). None of these strains aggregated under photoautotrophic conditions (Figure 3).

We conclude that suppressor mutations in the rev1 and rev4 strains almost completely restored the viability of the Δ*pilD* mutant in our standard laboratory conditions. The remaining two strains (rev2, rev3) were only partially complemented. Although the rev3 strain proliferated better than rev2 if both supplemented with glucose, it appeared phenotypically closest to the original Δ*pilD* mutant based on the poor accumulation of membrane proteins (Supplementary Figure 2), a compact cell aggregation (Figure 3), the growth inhibition, and loss of Chl during the prolonged cultivation on plates (Figure 2B) as well as in liquid cultures (Figure 2C).

### Effect of Suppressor Mutations on the Expression of *pilA1* Gene

**FIGURE 4:**
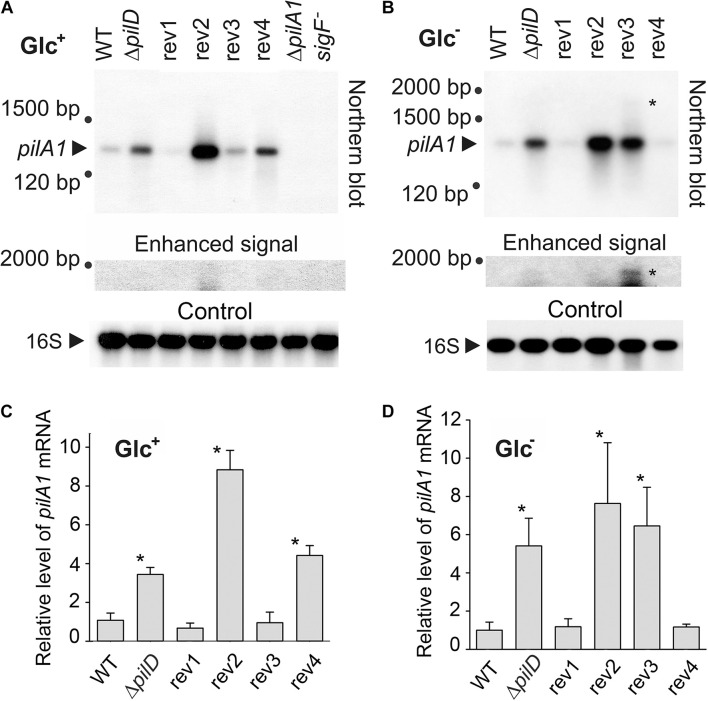
Cellular levels of *pilA1* mRNA in WT, Δ*pilD* mutant, and Δ*pilD* revertant strains. RNA was isolated from WT and mutant cells that were grown for 2 days in the presence **(A)** or absence **(B)** of glucose. RNA was blotted and hybridized sequentially with radiolabeled probes against *pilA1* mRNA and 16S rRNA. An unknown large transcript, detected with the *pilA1* probe, is indicated by an asterisk. As the signal of this long transcript is much weaker than the signal of *pilA1*, an enhanced image of the upper part of the membrane is also shown. Δ*pilA1*/*2* and *sigF*^–^ mutants were included as negative controls; 3.75 μg of total isolated RNA was loaded in each lane. **(C,D)** Hybridization was repeated using independent cell cultures, the density of the signals were quantified using ImageQuant TL 10 (GE Healthcare), normalized to the signal of 16S RNA and plotted. The values represent means ± SD from three independent measurements. Asterisks indicate statistically significant differences in the *pilA1* mRNA levels between WT and other strains as tested using a paired Student’s *t*-test (* *P* < 0.05).

As all suppressor mutations could potentially affect the expression of the *pilA1* gene, we quantified the *pilA1* transcripts by Northern blotting both in Glc^–^ and Glc^+^ conditions. Note, that the glucose itself had a strong suppressor effect on the prepilin toxicity (Figures 2A,B). The *pilA1* transcript was found to be monocistronic (Figures 4A,B), consistent with previous reports (He and Vermaas, 1999; Asayama and Imamura, 2008), and the *pilA1* mRNA was 4 to 5 times more abundant in the Δ*pilD* mutant both in Glc^–^ and Glc^+^ than in WT (Figures 4C,D). As expected no *pilA1* transcript was detected in the *sigF*^–^ mutant (Figure 4A).

In the rev1 strain, the H45P mutation in the SigF protein reduced the *pilA1* mRNA content to a similar level as in WT both in Glc^–^ and Glc^+^ conditions (Figure 4). On the other hand, in the rev4 strain (mutations in SigF and the γ subunit of RNAP) the *pilA1* transcript level dropped to the WT level only in Glc^–^ but remained similar as in the Δ*pilD* strain in mixotrophic conditions (Figure 4). The amount of *pilA1* mRNA in rev2 (mutation in the *pilA1* gene) was high (Figure 4), indicating that the suppressor effect of this revertant line is not due to the weakened *pilA1* expression. In the rev3 strain, the point mutation in the *pilA1-pilA2* intergenic region lowered the amount of *pilA1* transcript to the WT level only in Glc^+^ conditions. Interestingly, we also detected a longer *pilA1* transcript (∼1,800 bp) in rev3 and specifically under the photoautotrophic conditions (Figures 4A,B); this result was observed in three biological replicates (Supplementary Figure 3). The length of this transcript corresponds to *pilA1-pilA2-sll1696* polycistronic mRNA suggesting that the rev3 mutation might weaken the *pilA1* terminator.

Based on transcriptomic data, Kopf et al. (2014) identified a *Synechocystis* transcription unit (TU1331) coding for a putative *pilA1* anti-sense RNA with length ∼100 bp longer than the “sense” *pilA1* mRNA. The rev3 mutation is located in the promoter region of the expected anti-sense gene between −10 and −35 elements (Figure 1C). However, we did not detect such an antisense transcript on the Northern blot, which suggests that its level is much lower than that of *pilA1* (Figures 4A,B).

This transcript analysis revealed a complex regulation of the *pilA1* gene regarding the trophic mode. Since the pPilA1 protein is connected with lethality under Glc^–^ conditions (Figure 2B), the lowered *pilA1* transcript (to the level detected in WT; Figure 4D) in rev1 and rev4 after 48 h without glucose could explain the ability of these strains to grow photoautotrophically. On the other hand, the very strong expression of *pilA1* in the rev2 and rev3 strains in Glc^–^ (Figure 4D) implies that they suppress the prepilin toxicity by a different mechanism(s).

### Accumulation of PilA1 Prepilin and the Stability of Translocon Machinery

**FIGURE 5:**
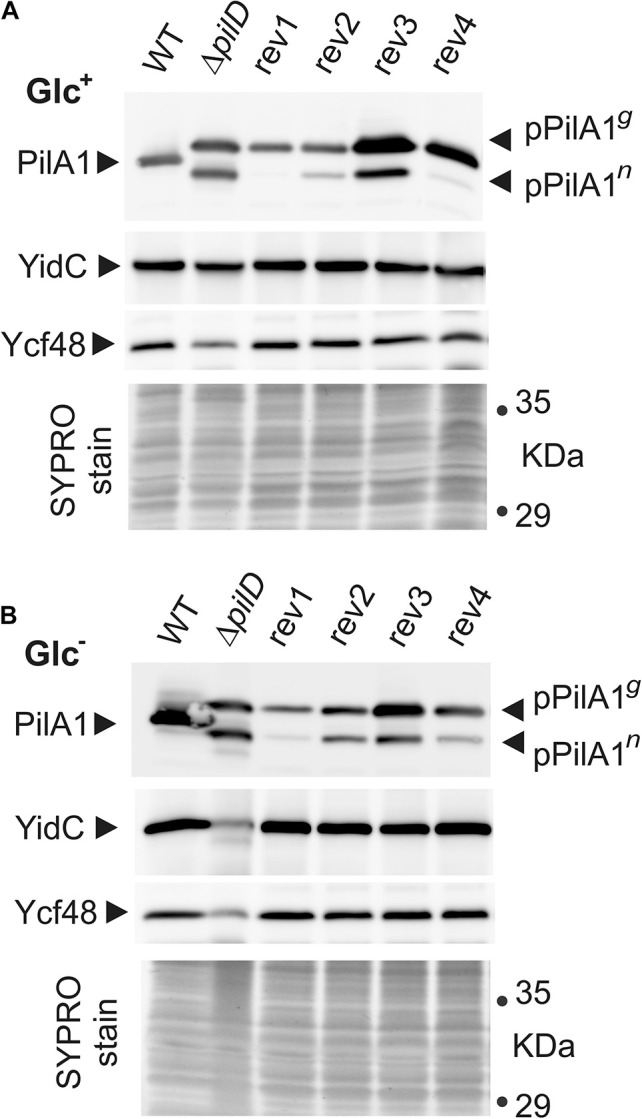
Cellular levels of pPilA1*^*g*^* and pPilA1*^*n*^* proteins and the stability of YidC and Ycf48 in the WT, Δ*pilD* mutant, and Δ*pilD* revertant strains. Membrane proteins were isolated from Glc^+^ **(A)** and Glc^–^ conditions **(B)**, separated by SDS-PAGE and blotted; the loading corresponded to the same number of cells based on OD_730_. Pilin and prepilin forms of PilA1, YidC insertase, and Ycf48 were immunodetected by specific antibodies. The gel stained with SYPRO Orange before blotting is shown below the blot as a loading control. pPilA1*^*g*^*, pPilA1*^*n*^* designate glycosylated and non-glycosylated prepilins, respectively.

The level of PilA1 protein in the isolated cellular membrane fraction was assessed using immunoblotting. The newly synthesized PilA1 protein (prepilin) is matured by the PilD protease and further glycosylated (Kim et al., 2009; Linhartová et al., 2014). In *Synechocystis* WT cells, we detected only the mature glycosylated PilA1 protein, whereas the prepilin or the non-glycosylated pilin were below the detection limits (Figure 5). It is notable that in WT cells the PilA1 content greatly increased after shifting cells from Glc^+^ to Glc^–^ conditions (Figure 5B and Supplementary Figure 4). This increase in the PilA1 level in WT has not been observed previously after 24 h of incubation under Glc^–^ conditions (Linhartová et al., 2014) indicating that the higher accumulation of PilA1 requires longer (48 h) incubation in the absence of glucose or it is stimulated with higher cell density.

Without processing protease in the Δ*pilD* strain, a high amount of prepilin accumulated both in Glc^+^ and Glc^–^ conditions, circa 50% of which was non-glycosylated pPilA1 (pPilA1*^*n*^*) and 50% glycosylated pPilA1 (pPilA1*^*g*^*; Figure 5). Consistently with the low level of *pilA1* mRNA, the rev1 cells contained almost no pPilA1*^*n*^* and less of pPilA1*^*g*^* than the Δ*pilD* mutant regardless of the growth conditions used. Similarly, low *pilA1* mRNA was accompanied by traces of pPilA1*^*n*^* and a low amount of pPilA1*^*g*^* in rev4 in autotropic conditions. In mixotrophic conditions, the enhanced *pilA1* transcripts of rev4 (Figure 4) were accompanied by a high amount of pPilA1*^*g*^* although the amount of pPilA1*^*n*^* remained low. Thus, *pilA* mRNA and prepilin protein levels correlated in suppressor lines containing mutations in the SigF factor unlike in the other two suppressor strains. The high *pilA1* transcript levels in rev2 strain did not lead to high pPilA1 content, rev2 contained less pPilA1 proteins than *pilD* mutant and especially pPilA1*^*n*^* content was low both in photoautotrophic and mixotrophic conditions (Figure 5). Unlike the other revertant strains, the rev3 strain contained more pPilA1*^*g*^* than the Δ*pilD* mutant both in photoautotrophic and mixotrophic conditions, whereas the level of pPilA1*^*n*^* in rev3 was lower than in Δ*pilD* mutant only in photoautotrophic conditions (Figure 5). Thus the ratio of pPilA1 forms in rev3 shifted from half glycosylated and half non-glycosylated in Glc^+^ to ∼75% of the glycosylated prepilin over ∼25% of the non-glycosylated in Glc^–^.

Accumulation of pPilA1 in the Δ*pilD* mutant in Glc^–^ was previously shown to cause degradation of the SecY translocase and the YidC insertase (Linhartová et al., 2014) as well the lumenal Ycf48 protein, which forms a complex with YidC and facilitates the insertion of Chl molecules into PSII subunits (Bučinská et al., 2018; Yu et al., 2018). This process was accompanied by abolished production of PSII subunits (Linhartová et al., 2014). The proteolytic degradation of YidC and Ycf48 in the Δ*pilD* mutant in Glc^–^ (Figure 5) is in agreement with the earlier results. Notably, the content and stability of YidC insertase and Ycf48 did not appear affected in any of the suppressor strains including rev3 that is deficient in the content of membrane proteins (Supplementary Figure 2).

### Synthesis and Glycosylation of pPilA1 Prepilin in Δ*pilD* Suppressor Strains

We have previously speculated that the pPilA1*^*n*^* form of prepilin, rather than the glycosylated pPilA1*^*g*^*, is responsible for the deleterious effect on the membrane-protein synthesis (Linhartová et al., 2014). In accordance with that, lower amounts of the pPilA1*^*n*^* (but not pPilA1*^*g*^*) than in *pilD* mutant was measured in all suppressor lines (Figure 5).

**FIGURE 6:**
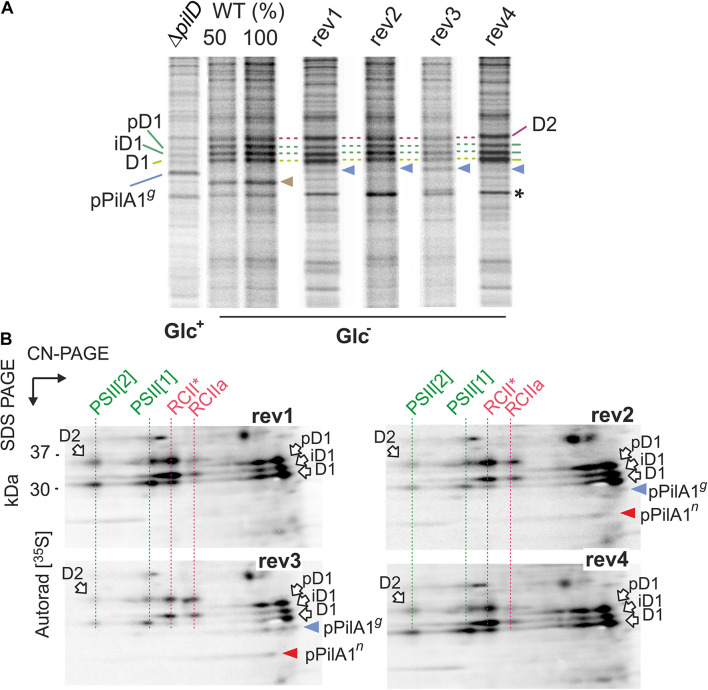
Synthesis of prepilin and PSII complexes in WT, Δ*pilD* mutant, and Δ*pilD* revertant strains. **(A)** WT and rev strains from Glc^–^ conditions and the Δ*pilD* (Glc^+^) were radiolabeled with a mixture of [^35^S]-Met/Cys using a 20-min pulse. Isolated membrane proteins were separated by 16–20% SDS-PAGE; 1 μg of Chl was loaded for each strain. The gels were stained with Coomassie Blue (Supplementary Figure 5), dried, and the labeled proteins were detected by phosphorimaging. The signal of pPilA*^*g*^* is indicated by a blue arrowhead and radiolabeled PilA1 by brown arrowhead. Asterisk indicates an intensively labeled band that has the same mobility in the gel as the pPilA*^*n*^*. All three forms of radiolabeled D1 subunit are marked by green dashed lines; pD1 and iD1 indicate unprocessed and partially processed forms of the D1 subunit. D2 subunit is marked by a purple dashed line. **(B)** The radiolabeled rev1-4 samples (4 μg of Chl) were separated by 2D CN/SDS-PAGE, stained by SYPRO Orange stain (see Supplementary Figure 6 for the stained gels) and blotted onto a PVDF membrane. The labeled proteins were detected by phosphorimaging and the signal of prepilins was verified by immunodetection (Supplementary Figure 7). Dashed red lines highlight PSII assembly intermediates RCIIa and RCII* (Yu et al., 2018), green lines highlight labeled PSII subunits assembled into monomeric and dimeric PSII. The signals of pPilA*^*g*^* and pPilA*^*n*^* are indicated by blue and red arrowheads, respectively.

To better understand prepilin toxicity, we assessed the synthesis of membrane proteins in cells grown under Glc^–^ conditions by radioactive pulse labeling (20 min) using a [^35^S]-Met/Cys mixture. Labeled membrane proteins were separated on SDS-PAGE (Coomassie and SYPRO Orange stained gels are shown in Supplementary Figures 5, 6) and autoradiographed (Figure 6). As shown previously, the synthesis of membrane proteins in the Δ*pilD* strain under Glc^–^ conditions is extremely weak (Linhartová et al., 2014) and we, therefore, compared the protein labeling of rev1-4 strains with WT (all after 48 h in Glc^–^) and with the Δ*pilD* (Glc^+^) sample. Intriguingly, even in a (fresh) Glc^+^ culture, the overall labeling of membrane proteins in Δ*pilD* was much less intensive than in WT (Glc^–^; Figure 6A). On the contrary, in rev1, rev2, and rev4 strains the total synthesis of membrane proteins was similar to that in WT. Consistent with the lowered amount of membrane proteins in rev3 in Glc^–^ conditions (Supplementary Figure 2), the total protein labeling in this strain was < 50% of that in WT including low synthesis of D1 and D2 core subunits of PSII (Figure 6B).

The radioactive signal on pPilA1*^*g*^* was much weaker in all rev strains than in the Δ*pilD* (Glc^+^) sample (Figure 6A). Given this low intensity, we cannot exclude a contribution of other weakly labeled proteins with gel mobility similar to pPilA1*^*g*^*. Moreover, the pPilA1*^*n*^* comigrates with an unknown intensively labeled protein (marked by an asterisk in Figure 6A) and therefore cannot be resolved on 1D-gel. To obtain a better resolution of labeled pPilA1*^*g*^* and pPilA1*^*n*^*, the identical samples as in Figure 6A were separated by 2D CN/SDS-PAGE and blotted. The radioactivity of the blotted proteins was visualized by phosphorimaging and the identity of pPilA1*^*g*^* and pPilA1*^*g*^* spots then verified by immunoblotting (Supplementary Figure 7).

We found that the synthesis of prepilin in rev1 and rev4 was slow as we were not able to detect the radiolabeled pPilA1*^*g*^* or pPilA1*^*n*^* in these strains (Figure 6B). Thus the low amount of both forms of pPilA1 protein detected by immunoblotting probably slowly accumulate and at least a fraction of pPilA1 is long-lived without the PilD protein in rev1 and rev4 (Figure 5). In contrast to rev1 and rev4, the pulse labeled forms of prepilin were well detectable in rev2 and rev3. In rev3, about a half of the labeled prepilins was non-glycosylated, whereas in rev2 pPilA1*^*n*^* was the prevailing form (Figure 6B). It should be noted that in the previous study we were not able to detect PilA1*^*n*^* in WT even by radiolabeling (Linhartová et al., 2014). Therefore, the glycosylation of PilA1 in WT must be very fast, but results from rev strains indicate that the glycosylation of prepilin occurs slowly.

Two-dimensional separation of radiolabeled proteins allowed us to inspect the process of PSII biogenesis. The labeled D1 and D2 subunits incorporated into the new PSII complexes were much weaker in rev3 than in other rev strains (Figure 6B). However, the process of PSII assembly was not restored in rev2 either. Although the synthesis of D1 and D2 subunits in rev2 appeared as intensive as in WT (Figure 6A), the signal of labeled D1 and D2 in monomeric and dimeric PSII was lower than in rev1 or rev4. The partially blocked biogenesis of PSII correlated with the lowered level of PSII in rev2 and rev3 (Figure 2D).

### The *pilA2-sll1696* Locus Promotes the Glycosylation of PilA1

**FIGURE 7:**
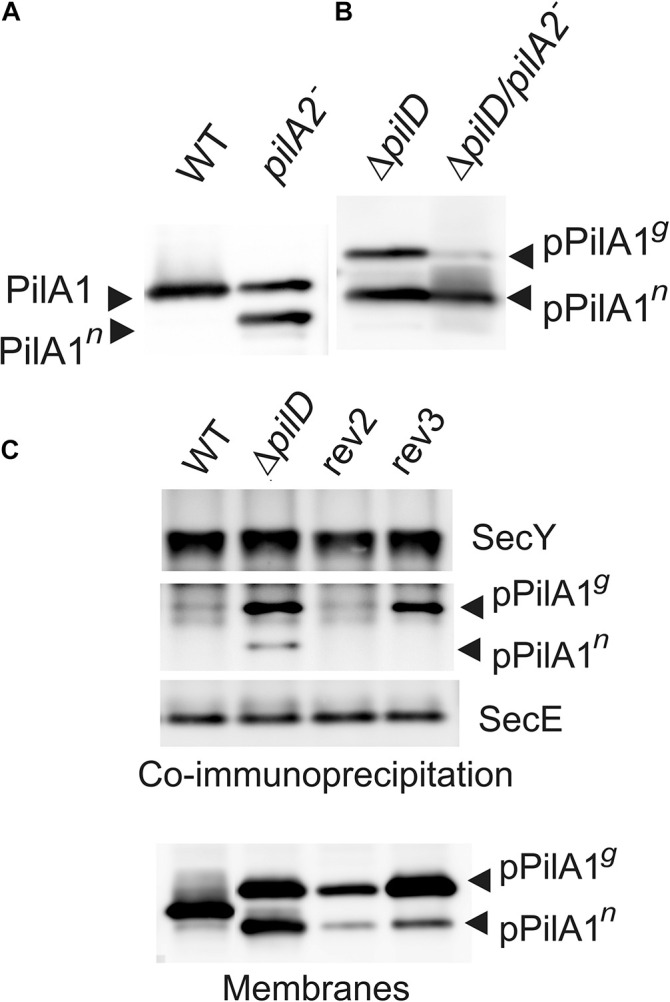
The role of *pilA2-sll1696* operon in the PilA1 biogenesis and the co-immunoprecipitation of the SecY translocase with prepilin. **(A)** Immunodetection of PilA1 in membrane proteins isolated from WT and *pilA2*^–^ cells grown under Glc^–^ conditions. Membrane proteins were separated by SDS-PAGE and blotted; PilA1*^*n*^* indicates a putative non-glycosylated form of PilA1. **(B)** Immunodetection of pPilA1 in separated membrane proteins from Δ*pilD* and Δ*pilD*/*pilA2*^–^ cells. Both strains were grown in Glc^+^ conditions. **(C)** The anti-SecY antibody was incubated with membrane proteins isolated from the WT, Δ*pilD* mutant and rev2 and rev3 strains grown in Glc^+^ conditions and then immobilized on Protein A—Sepharose (Sigma, Germany), eluted in SDS buffer, and separated by SDS-PAGE. The gel was blotted and probed with anti-SecY and anti-PilA1 antibodies; the anti-SecE antibody was re-probed as a control. The level of PilA1 forms is also shown for the identical membrane samples to those used for the co-immunoprecipitation.

The greatly reduced expression of *pilA1* in rev1 and rev4 under Glc^–^ offers a straightforward explanation of how the SigF and RNAP mutations suppress the loss of *pilD*. The mechanism(s) of rev2 and rev3 mutations, however, remained enigmatic. The rev3 mutation is located in the predicted terminator of the *pilA1* gene and might thus increase the expression of the downstream located *pilA2* and *sll1696* genes (Figure 1C); in WT these two genes are co-transcribed (Kopf et al., 2014). To see any potential effect of the disrupted *pilA2-sll1696* locus on the stability/biogenesis of PilA1 we immunodetected the PilA1 protein in *pilA2*^–^ (Glc^–^) and Δ*pilD*/*pilA2*^–^ (Glc^+^) strains. Interestingly, another form of PilA1 was detected in the *pilA2*^–^ strain, which would correspond to the non-glycosylated PilA1 (PilA1*^*n*^*; Figure 7A). Moreover, in the Δ*pilD*/*pilA2*^–^ strain, the glycosylated pPilA1*^*g*^* was almost completely absent while the level of pPilA1*^*n*^* remained high (Figure 7B). These data suggest that the *pilA2*-*sll1696* locus is required for the modification of (p)PilA1 and also support the model that, rather than the pPilA1*^*g*^*, the pPilA1*^*n*^* inhibits protein synthesis; note that the disruption of *pilA2* does not complement the Δ*pilD* mutant (Linhartová et al., 2014). Indeed, if compared to the original Δ*pilD* strain, the pPilA1*^*n*^* in rev3 (Glc^–^) appears specifically decreased (Figure 5B; see also Figure 7C).

### S3G Mutation in the Signal Peptide Weakens the Interaction of pPilA1 With the Membrane

The S3G suppressor mutation in rev2 is located in the signal peptide that is cleaved out by the PilD protease during the pPilA1 maturation. In our previous work, the native pPilA co-immunoprecipitated with SecY showing an aberrant interaction between the prepilins and the translocon (Linhartová et al., 2014). To check the potential effect of the S3G mutation of pilA1 on the interaction with translocons, we immunoprecipitated SecY and detected the co-purified pPilA1 in rev2 and rev3 strains. In the Δ*pilD* mutant the pPilA1*^*g*^* and a smaller amount of pPilA1*^*n*^* co-eluted with SecY but, importantly, the mutated pPilA1-S3G protein did not co-immuoprecipitated with SecY from the rev2 strain (Figure 7C). Although the pPilA1 was less abundant in rev2 membranes than in Δ*pilD* (Figure 7C), no detectable pPilA1 in the SecY precipitate suggested that the S3G mutation makes the association with translocons less likely. In contrast to rev2, the pPilA1*^*g*^* protein from the rev3 strain co-immunoprecipitated with SecY (Figure 7C).

**FIGURE 8:**
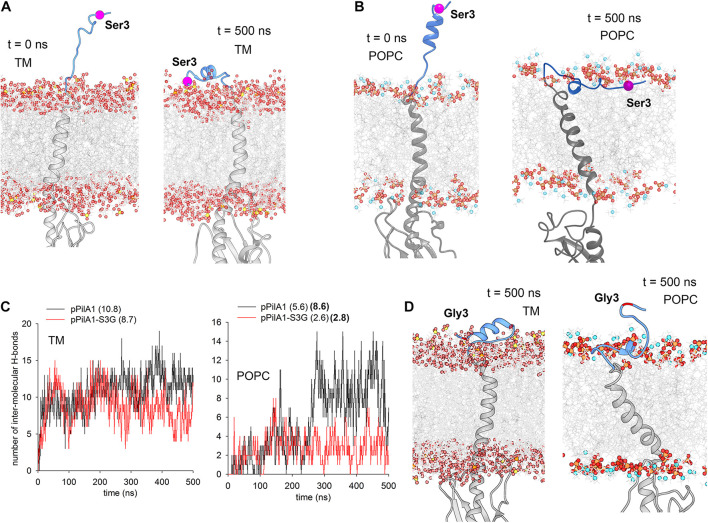
MD simulations of the *Synechocystis* pPilA1 and pPilA1-S3G proteins in a membrane bilayer. **(A)** Snapshots of the initial (0 ns) and the representative final (500 ns) conformation of pPilA1 in the TM (Supplementary Video 1). The signal peptide of pPilA1 is depicted in blue, the rest of the protein is in gray. Oxygen atoms in lipid molecules are shown as red spheres, sulfur (in sulfoquinovosyl diacylglycerol) as yellow spheres, phosphate atoms as orange spheres and nitrogen atoms (in POPC) as blue spheres. The position of Ser3 is highlighted by a magenta ball. Water molecules were omitted for clarity. **(B)** A snapshot of the initial (0 ns) and representative final (500 ns) positions of pPilA1 in the POPC bilayer (Supplementary Video 2). **(C)** Number of intermolecular hydrogen bonds between the pPilA1 signal peptide and TM or POPC lipid molecules for each frame (0–500 ns) of the MD simulation compared with the pPilA1-S3G. Numbers in parentheses indicate an average number of hydrogen bonds per frame formed during the simulation, numbers in bold show the number of hydrogen bonds for the period between 250–500 ns. See Supplementary Figures 8A,B for the description of hydrogen bonds between the lipid polar region and the pPilA1 signal peptide. **(D)** Representative final positions of PilA1-SG3 in the TM and POPC bilayer (Supplementary Videos 3, 4); see also Supplementary Figures 8C,D for the initial positions of the pPilA-S3G mutant protein.

According to available structures of the PilD-related aspartyl proteases (3S0X; 4HYC), the charged prepilin signal peptide is cleaved by PilD after being exposed to the cytosolic/stromal surface of the cell membrane (Hu et al., 2011; Li et al., 2013). To understand how the replacement of Ser3 with Gly3 in the signal peptide prevents the aberrant association of the prepilin to SecY, we performed MD simulations of the pPilA1*^*n*^* protein in a model TM (Daskalakis, 2018). As a starting position, the signal peptide was exposed in the cytosol (Figure 8A). Interestingly, the charged signal peptide almost immediately (6 ns) attached to the membrane surface, forming several hydrogen bonds between hydroxyl groups of galactolipids and Met1, Ser3, and Arg17 residues (Supplementary Figure 8A). Thereafter, the signal peptide remained attached to lipid sugars through the entire 0.5 μs MD simulation, just becoming deeply buried into the membrane (Figure 8A and Supplementary Video 1).

Although the association of the signal peptide with the model TM appears very robust, the SecY translocase is known to associate specifically with anionic lipids, such as phosphatidylglycerol or cardiolipin (reviewed in Crane and Randall, 2017). However, the total content of phosphatidylglycerol in *Synechocystis* cells is low (∼10%; Kopečná et al., 2015), and such a low content was used in our model TM (Daskalakis, 2018). To evaluate the effect of phospholipids on the interaction between the signal peptide and the membrane, we run the same simulation in the model phosphatidylcholine (POPC) membrane bilayer. In this case, the formation of stable contact between the signal peptide and POPC phosphate groups took longer (∼0.25 μs; Supplementary Figure 8B), but the peptide remained firmly embedded in the membrane later on (Figure 8B and Supplementary Video 2). Again, the network of hydrogen bonds between the signal peptide and lipids included the hydroxyl group of S3 residue (Supplementary Figure 8B).

We performed simulations of the mutated pPilA1-S3G and compared the strength of interaction between the signal peptide and lipids with the simulation of WT pPilA1 (see Supplementary Figures 8C,D for initial positions). In both TM and POPC membranes, the number of hydrogen bonds per frame was higher for the WT protein (Figure 8C). Notably, the interaction of the WT pPilA with the POPC membrane surface specifically strengthened (8.6 H-bonds per frame) after the formation of a stable contact with phospholipids. In contrast, the interaction of the mutated signal peptide with POPC remained transient (2.8 H-bonds per frame; Figure 8C) as documented also by the final position of the N-terminus extended above the membrane surface (Figure 8D and Supplementary Videos 3, 4).

## Discussion

The lack of PilD protease is lethal for *Synechocystis* cells grown under photoautotrophic conditions (Figure 2B), pPilA1 proteins accumulate in high quantity and the synthesis of membrane proteins is drastically reduced (Linhartová et al., 2014). In heterotrophic bacteria similar growth defects are not observed when PilD activity is inhibited (Pepe and Lory, 1998; Berry et al., 2019). Furthermore, the accumulation of other types of prepilins involved in adhesion (Tomich et al., 2006), secretion (Pugsley, 1993), or in the formation of T- and F-pili (Lai et al., 2002; Jain et al., 2011) do not appear to be detrimental for the bacterial cell.

In heterotrophic bacteria, Type IV pilins are synthesized and located in the plasma membrane before they are extracted from the membrane and assembled into long pili fibers (Craig et al., 2019). However, the *Synechocystis* major pilin PilA1 is not restricted to the plasma membrane and a significant fraction of cellular PilA1 can be detected in TM (Pisareva et al., 2011; Selão et al., 2016). Although it is likely that the presence of PilA1 in TMs results from a mistargeting, a functional role of pilin proteins in TM cannot be excluded. We propose that the synthesis and accumulation of major pilin in TM domains that are active in the biogenesis of photosynthetic apparatus could be the main reason why the elimination of PilD is not tolerated in *Synechocystis*. In our previous work, we hypothesized that the pPilA1 protein, and particularly its non-glycosylated pPilA1*^*n*^* form, jams the SecY-YidC holotranslocons thereby triggering their degradation (Linhartová et al., 2014). This would result in reduced synthesis of membrane proteins leading to the rapid decrease in the content of complexes with a very fast turning-over subunits, such as PSII (Figures 2, 6, 7).

**FIGURE 9:**
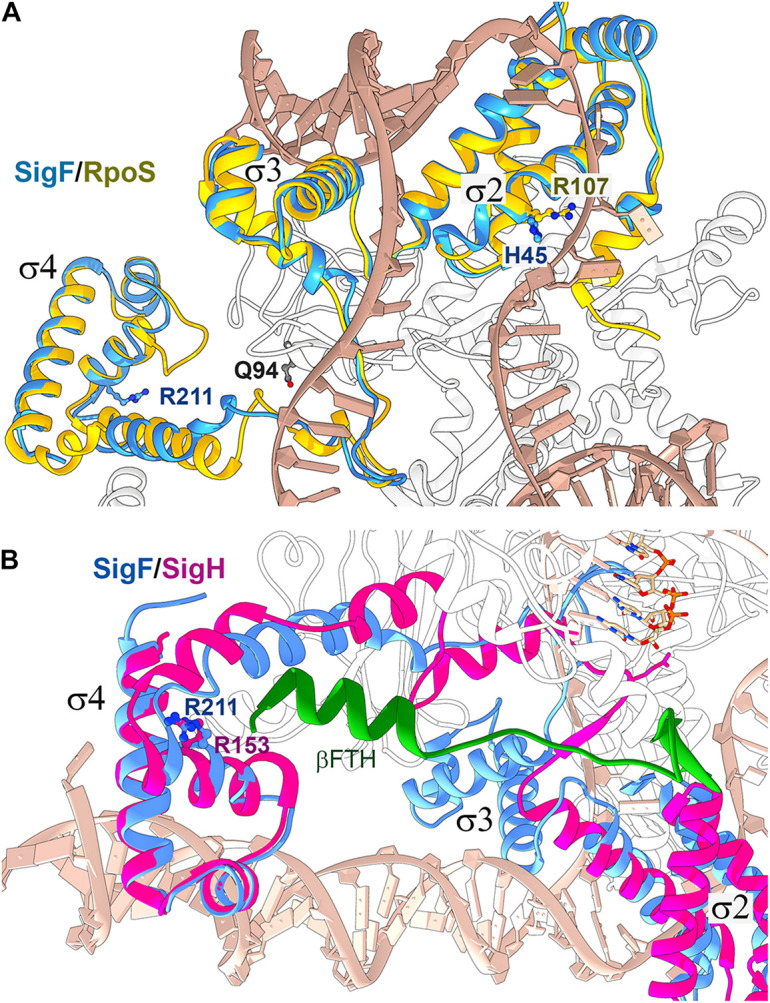
Structure alignment of the *Synechocystis* SigF with group 2 and ECF sigma factors bound in RNAP holoenzymes. **(A)** The structure of *Synechocystis* SigF was predicted using I-TASSER (C-score = 0.98; Yang et al., 2015) and aligned with the crystal structure of the transcription initiation complex from *E. coli* containing RpoS (PDB code 5IPL; Liu et al., 2016) using Chimera software (Pettersen et al., 2004). RpoS is depicted in gold, SigF in blue, DNA in tan and the RNAP β’ subunit in white. Residues in SigF that are mutated in rev1 and rev4 strains are shown in dark blue; Q94 corresponds to the E95 residue in the *Synechocystis* RNAP γ subunit. **(B)** The predicted structure of SigF was aligned with the crystal structure of the RNAP-SigH transcription initiation complex from *Mycobacterium tuberculosis* (PDB code 6DV9; Li et al., 2019). ECF sigma factors lack the σ3 domain, however, the SigF R211 residue mutated in rev4 is highly conserved in ECF sigma factors (R153 in SigH; Li et al., 2019). SigH is depicted in magenta, SigF in blue, RNAP subunit β in white and the C-terminal flap-tip helix of the RNAP-β subunit (βFTH) in green (see text for details).

In the present work, we analyzed four suppressor strains rescuing the lethal phenotype of the Δ*pilD* strain in photoautotrophic conditions. In rev1 and rev4 strains, the rescuing mechanism seems to be straightforward. The secondary mutations localize in the alternative SigF sigma factor (and rev4 contains an additional mutation in the γ subunit of RNAP) and highly reduce *pilA1* mRNA content compared to that in the parental Δ*pilD* strain (Figures 1, 4B). To understand how suppressor mutations actually act, we constructed a SigF model using the structure of *E. coli* RNAP-RpoS holoenzyme as a template (PDB code 5IPL; Liu et al., 2016) (Figure 9), because the structure of the cyanobacterial RNAP-SigF holoenzyme has not been resolved yet. The predicted structure of SigF resembles closely the structure of RpoS except for the N-terminal σ 1.2 domain of RpoS, which is missing in group 3 sigma factors to which the SigF belongs (see Srivastava et al., 2020) for a recent review. The histidine (H45) residue in SigF is located at the position of Arg or Lys conserved in most sigma factors including RpoS (Supplementary Figure 9). This particular R/K charged residue (R107) is in contact with the DNA chain two nucleotides before the -10 element (Figure 9; Liu et al., 2016). In contrast to the consensus cyanobacterial and *E. coli* -10 elements, the *pilA1* promoter contains a distinct -12 element (Asayama and Imamura, 2008). We, therefore, speculate that the H45P mutation impairs either the recognition of the *pilA1* promoter or the transition from closed to open promoter complex during transcription initiation.

The arginine residue R211 in RpoS (R211C mutation in rev4), conserved in all types of bacterial sigma factors, is located in the domain σ4 which binds to the −35 element. Structural data for the contacts between RpoS and the −35 element are not available; nonetheless, it has been solved recently for the extracytoplasmic function (ECF) sigma factors SigH (Li et al., 2019). Structural alignment between SigF and SigH suggests that the R211 residue does not interact with DNA but might provide contact with the RNAP core. In the rev4 strain, the other mutation is located in the γ subunit of the RNAP core, close to the predicted interaction site (Figure 9). Based on this *in silico* analysis, we propose that the rev4 mutations might lower the affinity of SigF to the RNAP core.

Due to the low *pilA1* mRNA content, rev1 and rev4 cells produced only low amounts of pPilA1, especially the most harmful pPilA1*^*n*^* form (Figure 5B) allowing normal membrane protein synthesis and the biogenesis of PSII (Figure 6B). It should be also noted that, despite the similar amount of *pilA1* mRNA in WT, rev1 and rev4 strains, the radiolabeling or the accumulation of pPilA1 is quite low in rev1 and rev4 strains when compared with the mature PilA1 protein in WT cells (Figures 5B, 6B). These data suggest that the fraction of the synthesized pPilA1 is quickly degraded, in line with our previous pulse-chase experiments (Linhartová et al., 2014).

Intriguingly, rev4 mutations show no effect on the *pilA1* mRNA level in the presence of glucose (Figure 4A). This finding implies an important effect of the trophic mode on the control over the *pilA1* expression and suggests that the formation of RNAP-SigF holoenzyme might be influenced by the tropic mode of *Synechocystis*. The suppressor effect of glucose on the lethality of *pilD* deletion is probably pleiotropic. The external source of glucose seems to downregulate the *pilA1* expression in the Δ*pilD* mutant (if judged from the ratio of *pilA1* mRNA in WT and *pilD*; Figure 4) and accessible glucose may promote the prepilin glycosylation to less harmful pPilA1*^*g*^* form. In addition, mixotrophic metabolism enables to better cope with the lower content of photosynthetic membrane proteins; note that the protein labeling in Δ*pilD* mutant cells is weak even under Glc^+^ conditions (Figure 6A).

Unlike in rev1 and rev4 strains, the *pilA1* mRNA is not reduced in rev2 and rev3 strains (Figure 4) and completely different molecular mechanisms were found to rescue the lethality of *pilD* deletion in these strains. Rev3 mutation is located in the *pilA1* terminator immediately upstream of the *pilA2-sll1696* gene pair that, even in WT, seems to be transcribed as a single unit with *pilA1* from the *pilA1* promoter (Kopf et al., 2014; Figure 1C). We however did not detect this long mRNA in WT, but only in rev3 strain (Supplementary Figure 3) implying that the rev3 mutation highly increases the level of the *pilA1-pilA2-sll1696* transcript. This result prompted us to check the effect of *pilA2-sll1696* inactivation on the PilA1 biosynthesis. In the *pilA2*^–^ mutant, non-glycosylated PilA1*^*n*^* accumulated and in the Δ*pilD*/*pilA2*^–^ double mutant the amount of glycosylated pPilA1*^*g*^* was low. The requirement of the PilA2 protein for the PilA1 glycosylation would be, however, unexpected since minor pilins have not been linked with the pilin glycosylation yet. Indeed, the inactivation of *pilA2* may affect the expression of the co-transcribed gene for Sll1696, which is annotated as a hypothetical protein and has homologs in many cyanobacteria. As genes for these homologs frequently map close to pilin genes, it is possible that the Sll1696, rather than PilA2, is required for the glycosylation of PilA1.

We suggest the rev3 mutation increases the level of *pilA2-sll1696* transcript thereby improving the rate of pPilA1 glycosylation. In rev3, immunodetection indicates that the majority of abundant pPilA1 was glycosylated (Figure 5) and in radiolabeling experiments about half of the pulse-labeled pPilA1 was glycosylated (Figure 6B). This was a specific feature in rev3 strain, whereas in the rev2 strain the pPilA1*^*g*^* form was hardly detectable in pulse labeling experiments (Figure 6B) and similar amounts of pPilA1*^*g*^* and pPilA1*^*n*^* forms were seen in immunoblots (Figure 5B). Altogether our results suggest that the non-glycosylated pPilA1*^*n*^* is more deleterious than the pPilA1*^*g*^* and the rescue of Δ*pilD* lethal phenotype in rev3 is suggested to be due to hyperefficient glycosylation of pPilA1. In *Neisseria gonorrhoeae*, the lack of glycosylation of the major pilin protein causes a growth arrest and authors speculated that the unassembled (non-glycosylated) pilin protein disrupts essential processes in the cell membrane (Vik et al., 2012).

The rev2 strain carries a secondary mutation in the signal peptide of pPilA1 protein. Our MD simulations show that the charged signal peptide of the native prepilin interacts tightly with the membrane surface (Figure 8) and the lateral diffusion of prepilin is likely much more restricted than that of the mutated prepilin or matured PilA1. As the mobility of membrane proteins can be facilitated by glycosylation (Hartel et al., 2016), we hypothesize that the pPilA1*^*n*^* has an even stronger tendency than the pPilA1*^*g*^* to accumulate in translocon-rich membrane domains. Nonetheless, the aberrant interaction between pPilA1 and SecY/YidC might not be so crucial to trigger translocon degradation as we proposed before (Linhartová et al., 2014). The pPilA1 from rev3 copurifies with SecY (Figure 7C) and, although the protein synthesis in this strain is impaired (Figure 6A), there is no sign of YidC degradation (Figure 5B). More likely, just the high concentration of prepilins in biogenic membrane zones can attenuate the activity of translocons, the proteolytic degradation may be detectable only after a very high degree of inactivation. The S3G mutation of pPilA1 should facilitate the mobility of pilin within the membrane, particularly in phospholipid-rich domains (Figure 8C). The pPilA1-S3G also seems to be less stable, which further improves the viability of the rev2 strain.

Strong aggregation of Δ*pilA1*/*2* and Δ*pilD* pilin mutants under mixotrophic conditions is an interesting phenotype with a potential value for cyanobacterial biotechnology. It is notable that in rev1 and rev4 strains, harboring mutations in *sigF*, the aggregation phenotype disappeared and the *sigF*^–^ strain also does not aggregate despite it lacks the PilA1 protein (Figure 1; Barker et al., 2006). Although the non-aggregation phenotype of *sigF*^–^ is inconsistent with the report of Flores et al. (2019), the authors admit that their Δ*sigF* mutant is derived from an aggregation-prone *Synechocystis* substrain. We can therefore conclude that the disrupted biogenesis of pilin proteins causes cell aggregation by a SigF-dependent mechanism, perhaps via secreted compounds (Flores et al., 2019).

## Data Availability Statement

The datasets presented in this study can be found in online repositories. The names of the repository/repositories and accession number(s) can be found below: https://www.ncbi.nlm.nih.gov/Traces/study/?acc=PRJNA757148&o=acc_s%3Aa.

## Author Contributions

ML, JKo, and RS were involved in the design of this study. ML, PS, KH, MT, JKo, JKn, JG, VG, TT, and RS performed and analyzed the data. ML, TT, and RS wrote the manuscript. All authors contributed to the article and approved the submitted version.

## Conflict of Interest

The authors declare that the research was conducted in the absence of any commercial or financial relationships that could be construed as a potential conflict of interest.

## Publisher’s Note

All claims expressed in this article are solely those of the authors and do not necessarily represent those of their affiliated organizations, or those of the publisher, the editors and the reviewers. Any product that may be evaluated in this article, or claim that may be made by its manufacturer, is not guaranteed or endorsed by the publisher.

## Footnotes

1
http://www.geneious.com

## References

[B1] Asayama M. Imamura S. (2008). Stringent promoter recognition and autoregulation by the group 3 sigma-factor SigF in the cyanobacterium *Synechocystis* sp. strain PCC 6803. *Nucleic Acids Res.* 36 5297–5305. 10.1093/nar/gkn453 18689440 PMC2532724

[B2] Barker M. De Vries R. Nield J. Komenda J. Nixon P. J. (2006). The Deg proteases protect *Synechocystis* sp PCC 6803 during heat and light stresses but are not essential for removal of damaged D1 protein during the photosystem two repair cycle. *J. Biol. Chem.* 281 30347–30355. 10.1074/jbc.M601064200 16912048

[B3] Bečková M. Gardian Z. Yu J. Koník P. Nixon P. J. Komenda J. (2017). Association of Psb28 and Psb27 proteins with PSII-PSI supercomplexes upon exposure of *Synechocystis* sp. PCC 6803 to high light. *Mol. Plant* 10 62–72. 10.1016/j.molp.2016.08.001 27530366

[B4] Berry J. L. Gurung I. Anonsen J. H. Spielman I. Harper E. Hall A. M. J. (2019). Global biochemical and structural analysis of the type IV pilus from the Gram-positive bacterium *Streptococcus sanguinis*. *J. Biol. Chem.* 294 6796–6808. 10.1074/jbc.RA118.006917 30837269 PMC6497953

[B5] Bhaya D. Bianco N. R. Bryant D. Grossman A. (2000). Type IV pilus biogenesis and motility in the cyanobacterium *Synechocys*tis sp. PCC 6803. *Mol. Microbiol.* 37 941–951. 10.1046/j.1365-2958.2000.02068.x 10972813

[B6] Bowers K. J. Chow E. Xu H. Dror R. O. Eastwood M. P. Gregersen B. A. (2006). *Scalable algorithms for molecular dynamics simulations on commodity clusters in International conference for high performance computing, networking, storage and analysis*: *Machinery.* New York, NY.

[B7] Bučinská L. Kiss E. Koník P. Knoppová J. Komenda J. Sobotka R. (2018). The ribosome-bound protein Pam68 promotes insertion of Chl into the CP47 subunit of photosystem II. *Plant Physiol.* 176 2931–2942. 10.1104/pp.18.00061 29463774 PMC5884600

[B8] Conradi F. D. Zhou R. Q. Oeser S. Schuergers N. Wilde A. Mullineaux C. W. (2019). Factors controlling floc formation and structure in the cyanobacterium *Synechocystis* sp. strain PCC 6803. *J. Bacteriol.* 201 e319–e344. 10.1128/jb.00344-19 31262837 PMC6755745

[B9] Craig L. Forest K. T. Maier B. (2019). Type IV pili: dynamics, biophysics and functional consequences. *Nat. Rev. Microbiol.* 17 429–440. 10.1038/s41579-019-0195-4 30988511

[B10] Crane J. M. Randall L. L. (2017). The Sec system: protein export in *Escherichia coli*. *EcoSal. Plus* 7:17. 10.1128/ecosalplus.ESP-0002-2017 29165233 PMC5807066

[B11] Daskalakis V. (2018). Protein-protein interactions within photosystem II under photoprotection: the synergy between CP29 minor antenna, subunit S (PsbS) and zeaxanthin at all-atom resolution. *Phys. Chem. Chem. Phys.* 20 11843–11855. 10.1039/c8cp01226a 29658553

[B12] Dobáková M. Sobotka R. Tichý M. Komenda J. (2009). Psb28 protein is involved in the biogenesis of the photosystem II inner antenna CP47 (PsbB) in the cyanobacterium *Synechocystis* sp. PCC 6803. *Plant Physiol.* 149 1076–1086. 10.1104/pp.108.130039 19036835 PMC2633855

[B13] Duan Y. Wu C. Chowdhury S. Lee M. C. Xiong G. Zhang W. (2003). A point-charge force field for molecular mechanics simulations of proteins based on condensed-phase quantum mechanical calculations. *J. Comput. Chem.* 24 1999–2012. 10.1002/jcc.10349 14531054

[B14] Ermakova-Gerdes S. Vermaas W. F. J. (1999). Inactivation of the open reading frame *slr0399* in *Synechocystis* sp. PCC 6803 functionally complements mutations near the Q(A) niche of photosystem II. A possible role of Slr0399 as a chaperone for quinone binding. *J. Biol. Chem.* 274 30540–30549. 10.1074/jbc.274.43.30540 10521436

[B15] Flores C. Santos M. Pereira S. B. Mota R. Rossi F. De Philippis R. (2019). The alternative sigma factor SigF is a key player in the control of secretion mechanisms in *Synechocysti*s sp. PCC 6803. *Environ. Microbiol.* 21 343–359. 10.1111/1462-2920.14465 30394639

[B16] Giltner C. L. Nguyen Y. Burrows L. L. (2012). Type IV pilin proteins: versatile molecular modules. *Micro Mol. Biol. Rev.* 76 740–772. 10.1128/mmbr.00035-12 23204365 PMC3510520

[B17] Goosens V. J. Busch A. Georgiadou M. Castagnini M. Forest K. T. Waksman G. (2017). Reconstitution of a minimal machinery capable of assembling periplasmic type IV pili. *Proc. Natl. Acad. Sci. USA* 114 E4978–E4986. 10.1073/pnas.1618539114 28588140 PMC5488919

[B18] Hartel A. J. Glogger M. Jones N. G. Abuillan W. Batram C. Hermann A. (2016). N-glycosylation enables high lateral mobility of GPI-anchored proteins at a molecular crowding threshold. *Nat. Commun.* 7:12870. 10.1038/ncomms12870 27641538 PMC5031801

[B19] He Q. Vermaas W. F. J. (1999). Genetic deletion of proteins resembling type IV pilins in Synechocystis sp. PCC 6803: their role in binding or transfer of newly synthesized chlorophyll. *Plant Mol. Biol.* 39 1175–1188. 10.1023/a:1006177103225 10380804

[B20] Hu J. Xue Y. Lee S. Ha Y. (2011). The crystal structure of GXGD membrane protease FlaK. *Nature* 475 528–531. 10.1038/nature10218 21765428 PMC3894692

[B21] Jain S. Kahnt J. Van Der Does C. (2011). Processing and maturation of the pilin of the type IV secretion system encoded within the gonococcal genetic island. *J. Biol. Chem.* 286 43601–43610. 10.1074/jbc.M111.264028 22006923 PMC3243527

[B22] Kim Y. H. Kim J. Y. Kim S. Y. Lee J. H. Lee J. S. Chung Y. H. (2009). Alteration in the glycan pattern of pilin in a nonmotile mutant of *Synechocystis* sp. PCC 6803. *Proteomics* 9 1075–1086. 10.1002/pmic.200800372 19180537

[B23] Komenda J. Krynická V. Zakar T. (2019). Isolation of thylakoid membranes from the cyanobacterium *Synechocystis* sp. PCC 6803 and analysis of their photosynthetic pigment-protein complexes by clear native-PAGE. *Bio-Protocol* 9:e3126. 10.21769/BioProtoc.3126 33654759 PMC7854132

[B24] Kopečná J. Pilný J. Krynická V. Tomčala A. Kis M. Gombos Z. (2015). Lack of phosphatidylglycerol inhibits Chl biosynthesis at multiple sites and limits Chlide reutilization in the cyanobacterium *Synechocystis* 6803. *Plant Physiol.* 169 1307–1317. 10.1104/pp.15.01150 26269547 PMC4587476

[B25] Kopf M. Klähn S. Scholz I. Matthiessen J. K. F. Hess W. R. Voß B. (2014). Comparative analysis of the primary transcriptome of *Synechocys*tis sp. PCC 6803. *DNA Res.* 21 527–539. 10.1093/dnares/dsu018 24935866 PMC4195498

[B26] Lai E. M. Eisenbrandt R. Kalkum M. Lanka E. Kado C. I. (2002). Biogenesis of T pili in *Agrobacterium tumefaciens* requires precise VirB2 propilin cleavage and cyclization. *J. Bacteriol.* 184 327–330. 10.1128/jb.184.1.327-330.2002 11741876 PMC134783

[B27] LaPointe C. F. Taylor R. K. (2000). The type 4 prepilin peptidases comprise a novel family of aspartic acid proteases. *J. Biol. Chem.* 275 1502–1510. 10.1074/jbc.275.2.1502 10625704

[B28] Li L. Fang C. Zhuang N. Wang T. Zhang Y. (2019). Structural basis for transcription initiation by bacterial ECF sigma factors. *Nat. Commun.* 10:1153. 10.1038/s41467-019-09096-y 30858373 PMC6411747

[B29] Li X. Dang S. Yan C. Gong X. Wang J. Shi Y. (2013). Structure of a presenilin family intramembrane aspartate protease. *Nature* 493 56–61. 10.1038/nature11801 23254940

[B30] Linhartová M. Bučinská L. Halada P. Ječmen T. Šetlík J. Komenda J. (2014). Accumulation of the Type IV prepilin triggers degradation of SecY and YidC and inhibits synthesis of Photosystem II proteins in the cyanobacterium *Synechocystis* PCC 6803. *Mol. Microbiol.* 93 1207–1223. 10.1111/mmi.12730 25060824

[B31] Liu B. Zuo Y. Steitz T. A. (2016). Structures of *E. coli* sigmaS-transcription initiation complexes provide new insights into polymerase mechanism. *Proc. Natl. Acad. Sci. USA* 113 4051–4056. 10.1073/pnas.1520555113 27035955 PMC4839411

[B32] Marceau M. Forest K. Béretti J.-L. Tainer J. Nassif X. (1998). Consequences of the loss of O-linked glycosylation of meningococcal type IV pilin on piliation and pilus-mediated adhesion. *Mol. Microbiol.* 27 705–715. 10.1046/j.1365-2958.1998.00706.x 9515697

[B33] Pepe J. C. Lory S. (1998). Amino acid substitutions in PilD, a bifunctional enzyme of *Pseudomonas aeruginosa*: Effect on leader peptidase and n-methyltransferase activities in vitro and in vivo. *J. Biol. Chem.* 273 19120–19129. 10.1074/jbc.273.30.19120 9668097

[B34] Pettersen E. F. Goddard T. D. Huang C. C. Couch G. S. Greenblatt D. M. Meng E. C. (2004). UCSF Chimera–a visualization system for exploratory research and analysis. *J. Comput. Chem.* 25 1605–1612. 10.1002/jcc.20084 15264254

[B35] Pisareva T. Kwon J. Oh J. Kim S. Ge C. R. Wieslander A. (2011). Model for membrane organization and protein sorting in the cyanobacterium *Synechocystis* sp PCC 6803 inferred from proteomics and multivariate sequence analyses. *J. Proteome. Res.* 10 3617–3631. 10.1021/pr200268r 21648951

[B36] Porra R. J. Thompson W. A. Kriedemann P. E. (1989). Determination of accurate extinction coefficients and simultaneous equations for assaying Chls *a* and *b* extracted with four different solvents: verification of the concentration of Chl standards by atomic absorption spectroscopy. *Biochim. Biophys. Acta* 975 384–394. 10.1016/S0005-2728(89)80347-0

[B37] Pugsley A. P. (1993). Processing and methylation of PulG, a pilin-like component of the general secretory pathway of *Klebsiella oxytoca*. *Mol. Microbiol.* 9 295–308. 10.1111/j.1365-2958.1993.tb01691.x 8412682

[B38] Sachelaru I. Winter L. Knyazev D. G. Zimmermann M. Vogt A. Kuttner R. (2017). YidC and SecYEG form a heterotetrameric protein translocation channel. *Sci. Rep.* 7:101. 10.1038/s41598-017-00109-8 28273911 PMC5427846

[B39] Schuergers N. Wilde A. (2015). Appendages of the cyanobacterial cell. *Life* 5 700–715. 10.3390/life5010700 25749611 PMC4390875

[B40] Selão T. T. Zhang L. Knoppová J. Komenda J. Norling B. (2016). Photosystem II assembly steps take place in the thylakoid membrane of the cyanobacterium *Synechocyst*is sp. PCC6803. *Plant Cell Physiol.* 57 95–104. 10.1093/pcp/pcv178 26578692

[B41] Srivastava A. Summers M. L. Sobotka R. (2020). Cyanobacterial sigma factors: Current and future applications for biotechnological advances. *Biotechnol. Adv.* 40:107517. 10.1016/j.biotechadv.2020.107517 31945415

[B42] Strom M. S. Nunn D. N. Lory S. (1993). A single bifunctional enzyme, PilD, catalyzes cleavage and N-methylation of proteins belonging to the type IV pilin family. *Proc. Nat. Acad. Sci. USA* 90 2404–2408. 10.1073/pnas.90.6.2404 8096341 PMC46095

[B43] Tajima N. Sato S. Maruyama F. Kaneko T. Sasaki N. V. Kurokawa K. (2011). Genomic structure of the cyanobacterium *Synechocystis* sp. PCC 6803 strain GT-S. *DNA Res.* 18 393–399. 10.1093/dnares/dsr026 21803841 PMC3190959

[B44] Tichý M. Bečková M. Kopečná J. Noda J. Sobotka R. Komenda J. (2016). Strain of *Synechocystis* PCC 6803 with aberrant assembly of photosystem II contains tandem duplication of a large chromosomal region. *Front. Plant Sci.* 7:648. 10.3389/fpls.2016.00648 27242849 PMC4867675

[B45] Tomich M. Fine D. H. Figurski D. H. (2006). The TadV protein of *Actinobacillus actinomycetemcomitans* is a novel aspartic acid prepilin peptidase required for maturation of the Flp1 pilin and TadE and TadF pseudopilins. *J. Bacteriol.* 188 6899–6914. 10.1128/JB.00690-06 16980493 PMC1595517

[B46] Tyystjärvi T. Herranen M. Aro E. M. (2001). Regulation of translation elongation in cyanobacteria: membrane targeting of the ribosome nascent−chain complexes controls the synthesis of D1 protein. *Mol. Microbiol.* 40 476–484. 10.1046/j.1365-2958.2001.02402.x 11309129

[B47] Vik A. Aspholm M. Anonsen J. H. Borud B. Roos N. Koomey M. (2012). Insights into type IV pilus biogenesis and dynamics from genetic analysis of a C-terminally tagged pilin: a role for O-linked glycosylation. *Mol. Microbiol.* 85 1166–1178. 10.1111/j.1365-2958.2012.08166.x 22882659

[B48] Yang J. Yan R. Roy A. Xu D. Poisson J. Zhang Y. (2015). The I-TASSER Suite: protein structure and function prediction. *Nat. Methods* 12 7–8. 10.1038/nmeth.3213 25549265 PMC4428668

[B49] Yu J. Knoppová J. Michoux F. Bialek W. Cota E. Shukla M. K. (2018). Ycf48 involved in the biogenesis of the oxygen-evolving photosystem II complex is a seven-bladed beta-propeller protein. *Proc. Natl. Acad. Sci. USA* 115 E7824–E7833. 10.1073/pnas.1800609115 30061392 PMC6099905

