# Supplementary material for: Mutations Suppressing the Lack of Prepilin Peptidase Provide Insights Into the Maturation of the Major Pilin Protein in Cyanobacteria
Source: Front Microbiol. 2021 Oct 12;12:756912. doi: 10.3389/fmicb.2021.756912 (PMC8546353; doi:10.3389/fmicb.2021.756912)
Supplement: Supplementary file 1 [file Data_Sheet_1.pdf]

SUPPLEMENTAL INFORMATION

Mutations suppressing the lack of prepilin peptidase provide insights into the maturation of the major pilin protein in cyanobacteria

Markéta Linhartová, Petra Skotnicová, Kaisa Hakkila, Martin Tichý, Josef Komenda, Jana Knoppová, Joan F. Gilabert, Victor Guallar, Taina Tyystjärvi, and Roman Sobotka

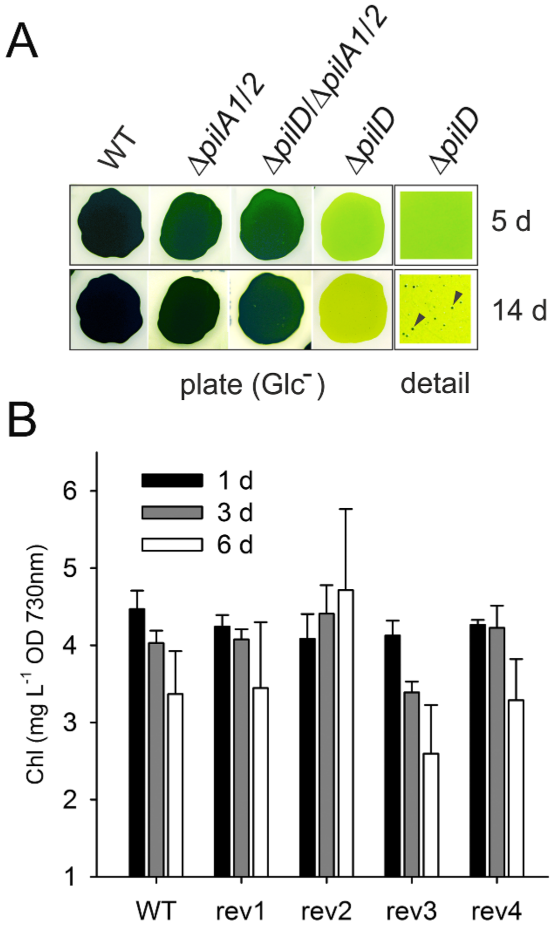

Figure S1. **Generation of Δ*pilD* suppressor mutations and chlorophyll (Chl) content in suppressor strains.** A) The originally isolated Δ*pilD* mutants together with the WT, Δ*pilA1/2* and, Δ*pilD*/Δ*pilA1/2* strain (Linhartová et al. 2014) were streaked onto BG-11 plate and cultivated under mixotrophic conditions. Pictures of the strains were taken on the 5th (5 d) and 14th (14 d) day of cultivation; arrowheads indicate suppressor mutants. B) Cellular Chl content was measured during photoautotrophic cultivation of WT and rev1-4 strains (see also Fig. 2B). The Chl content was measured spectrophotometrically at days 1, 3, and 6 and normalized to an optical density at 730 nm.

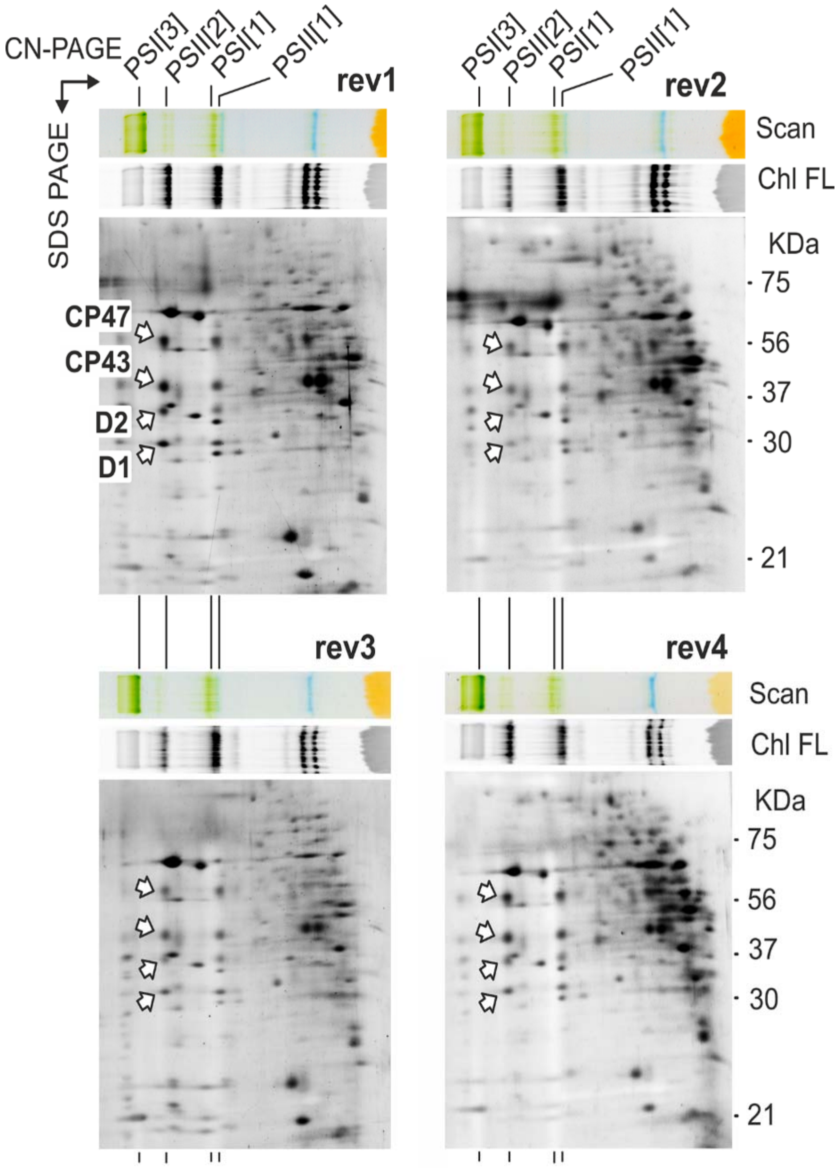

Figure S2. 2D CN/SDS-PAGE of membrane protein complexes isolated from Δ*pilD* suppressor strains. Suppressor strains (rev1-4) were grown for 48 h in the absence of Glc. Isolated membranes were solubilized and separated by CN-PAGE on the same gel (see Fig. 2C), 4 µg of Chl was loaded for each strain. After separation, the gel was scanned and PSII complexes were detected by Chl fluorescence after excitation by blue light in LAS 4000 (Fuji). The gel stripes were cut individually and separated in pairs (rev1 and rev2, rev3 and rev4) in the same gels in the second dimension by 12-20 % SDS-PAGE. The gel was stained with SYPRO Orange; core large subunits of PSII in dimeric complexes are indicated. PSI[3] is the trimer of PSI, PSI[1] is the monomer of PSI, PSII[2] is the dimer of PSII and PSII[1] is the monomer of PSII. White arrows indicate CP47, CP43, D2, and D1 subunits of PSII.

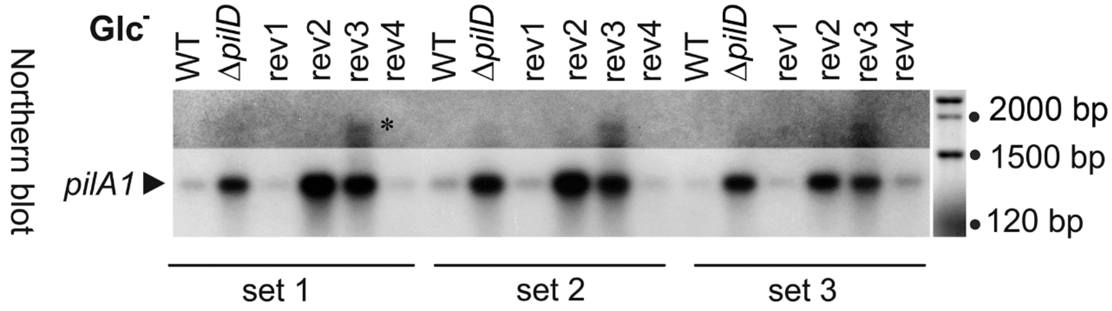

Figure S3. **Cellular levels of *pilA1* mRNA in the Δ*pilD* revertant strains.** A) RNA was isolated from WT and mutant cells which were grown for 2 days in the absence of glucose (Glc$^-$); sets 1-3 represent three independent biological replicates. RNA was blotted and hybridized with a radiolabeled probe against *pilA1* mRNA. The intensity of the upper part of the radiogram was enhanced to visualize the signal of a longer RNA molecule (marked by asterisk) hybridizing with the *pilA1* probe. See Fig. 4 for further details.

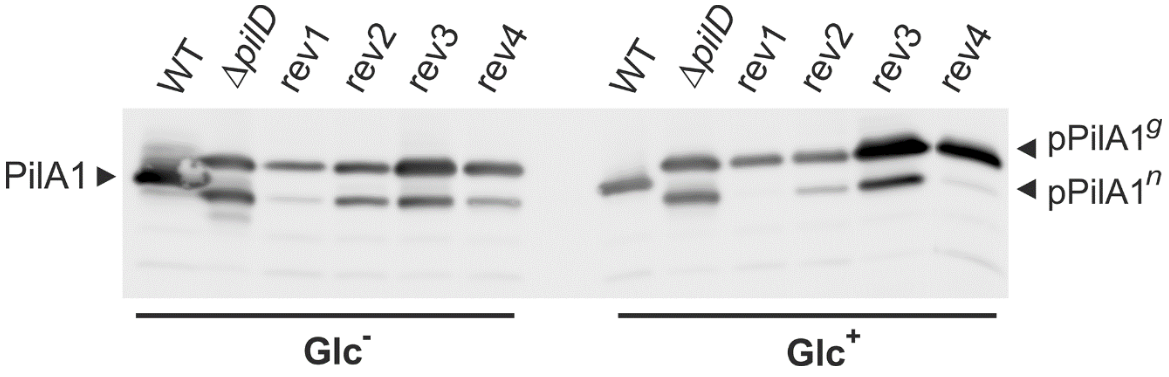

Figure S4. **Cellular levels of PilA1, pPilA1*g*, and pPilA1*n* proteins under photoautotrophic (Glc-) and mixotrophic (Glc+) conditions.** Isolated membrane proteins were separated by SDS-PAGE and blotted; the loading corresponded to the same number of cells based on OD_{730}. Pilin and prepilin forms of PilA1were immunodetected by specific antibodies; pPilA1*g*, pPilA1*n* designate glycosylated and non-glycosylated prepilins, respectively. See Fig. 5 for further details.

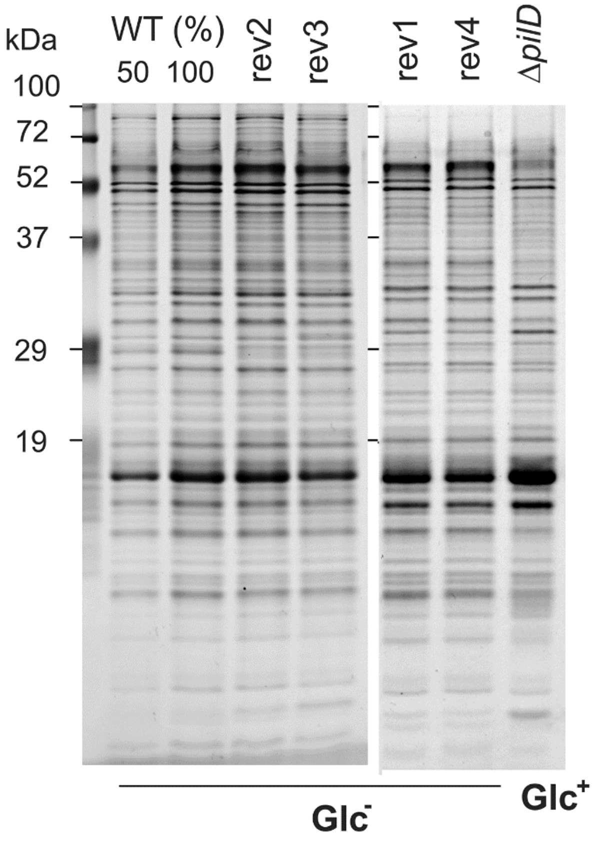

Figure S5. **SDS-PAGE of membrane proteins isolated from WT, the Δ*pilD* mutant, and the Δ*pilD* suppressor strains**. All strains were grown under Glc⁻ conditions except *pilD* that was grown mixotrophically. Cells were radiolabeled with a mixture of [^{35}S]-Met/Cys using a 20-min pulse (see Fig. 6A). Isolated membranes were solubilized and proteins corresponding to 1 µg of Chl were separated in 16-20 % SDS-PAGE gels. The gel was stained with Coomassie blue. Please note that the order of samples is different than is presented in Fig. 6A.

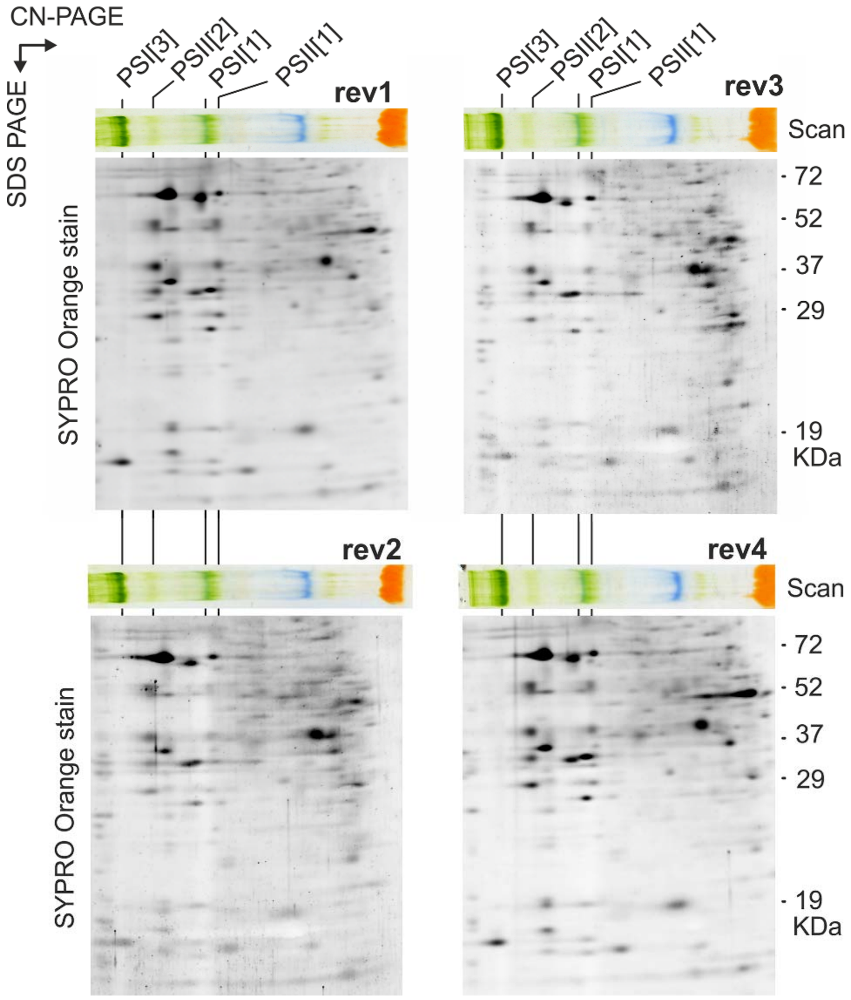

Figure S6. 2D CN/SDS-PAGE of membrane proteins isolated from the radiolabelled Δ*pilD* suppressor strains. Cells, grown under Glc$^-$ conditions, were radiolabeled with a mixture of [^{35}S]-Met/Cys using a 20-min pulse (see Fig. 6B). Isolated membranes were solubilized and separated by CN-PAGE; 4 µg of Chl was loaded for each strain. The gel stripes from the first dimension were cut and separated in the second dimension on two 16-20 % SDS-PAGE gels, each combining a pair of stripes (rev1 and rev4, rev2 and rev3). Gels were stained with SYPRO Orange. PSI[3] is the trimer of PSI, PSI[1] is the monomer of Photosystem I, PSII[2] is the dimer of PSII and PSII[1] is the monomer of PSII.

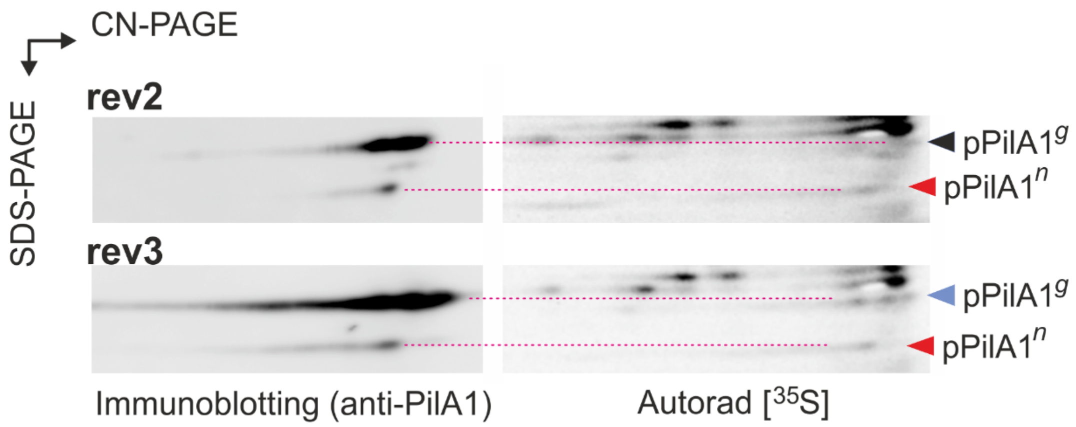

Figure S7. **Synthesis and accumulation of PilA1 prepilin in rev2 and rev3 suppressor strains.** The radiolabelled rev2 and rev3 samples (4 µg of Chl) were separated by 2D CN/SDS-PAGE, stained by SYPRO Orange stain (see supplementary Fig. S6 for the stained gel) and blotted onto a PVDF membrane. The labelled proteins were first detected by phosphorimaging (Fig. 6B) and then PilA1 prepilins were immunodetected by specific antibodies. Signals of radiolabelled and immunodetected PilA1 prepilin forms are aligned (dashed lines).

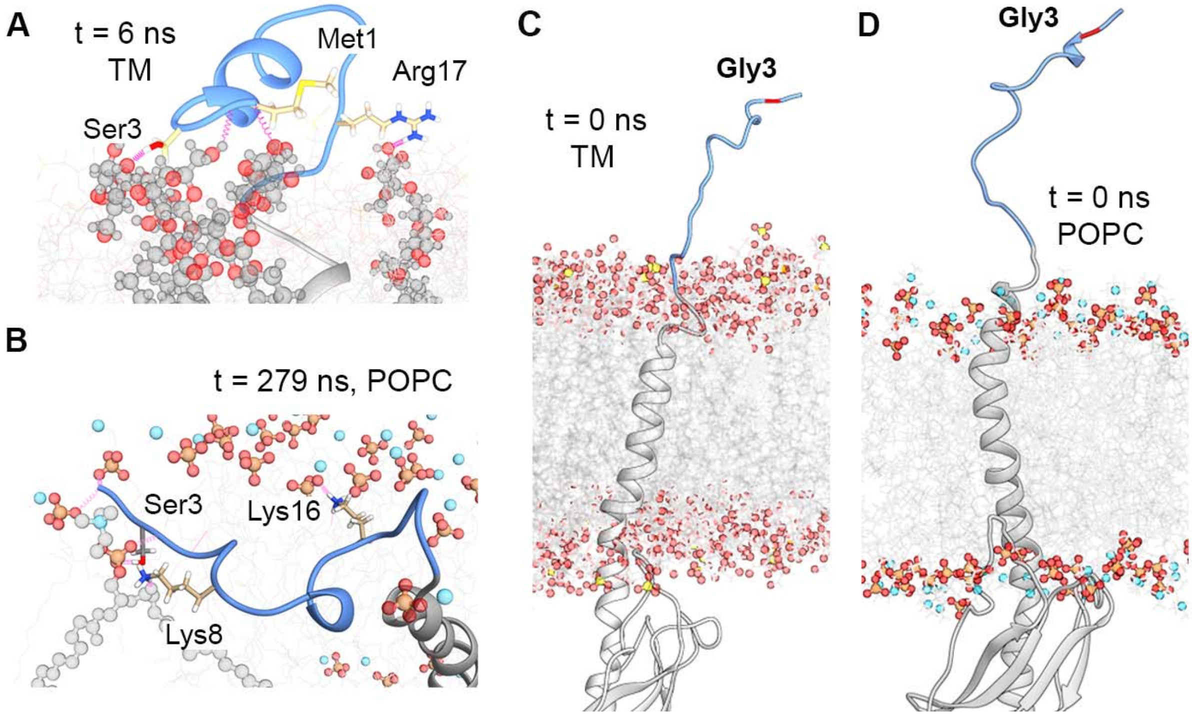

Figure S8. **MD simulations of the *Synechocystis* pPilA1 and pPilA1-S3G proteins in membrane bilayers**. A) A snapshot (6 ns) showing the first stable interaction between the pPilA1 signal peptide and the lipid polar region of TM; established hydrogen bonds are highlighted as purple springs. B) A first stable interaction (279 ns) between pPilA1 signal peptide and POPC lipids. C) and D) Snapshots of the initial (0 ns) conformation of pPilA-S3G in the thylakoid membrane (TM) and the POPC membrane.

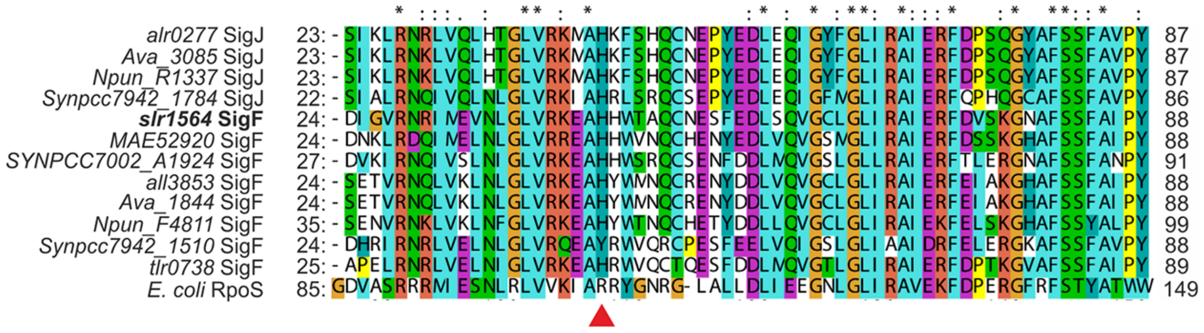

Figure S9. Amino-acid sequence alignment of the N-terminal part of cyanobacterial group 3 sigma factors (SigF and SigJ). The red triangle shows the position of the rev1 mutation in *Synechocystis* SigF (*slr1564*). The asterisks designate fully conserved amino acid residues and double and single dots strongly and weakly-conserved residues, respectively *E. coli* RpoS is included as an example of group 2 sigma factors. Gene sources: *alr0277, all3853* - *Anabaena* PCC 7120; *ava_3085*, *ava_1844* - *Anabaena variabilis* ATCC 29413; *Npun_R1337*, *Npun_F4811* - *Nostoc punctiforme* ATCC 29133; *Synpcc7942_1784*, *Synpcc7942_1510* - *Synechococcus elongatus* PCC 7942; *MAE52920* - *Microcystis aeruginosa* NIES-843; *SYNPCC7002_A1924* - *Synechococcus elongatus* PCC 7002; *tlr0738* - *Thermosynechococcus elongatus* BP-1.

SUPPLEMENTARY TABLE

Table S1. A list of oligonucleotides used in this study.

**Oligonucleotide**	**Sequence**
pilA1-f	5’-GACAATCATATGGCTAGTAATTTTAAATTC-3’
pilA1-r	5’-GACAATGCTAGCTTTAATTACTTCAGCACCAC-3’
rrn-f	5’-AGCGTCCGTAGGTGGTTATG-3’
rrn-r	5’-CACATACTCCACCGCTTGTG-3

References

Linhartová, M., Bučinská, L., Halada, P., Ječmen, T., Šetlík, J., Komenda, J., and Sobotka, R. (2014). Accumulation of the Type IV prepilin triggers degradation of SecY and YidC and inhibits synthesis of Photosystem II proteins in the cyanobacterium *Synechocystis* PCC 6803. Mol Microbiol **93:** 1207-1223.
